# Effects of fin fold mesenchyme ablation on fin development in zebrafish

**DOI:** 10.1371/journal.pone.0192500

**Published:** 2018-02-08

**Authors:** Robert L. Lalonde, Marie-Andrée Akimenko

**Affiliations:** Department of Biology, University of Ottawa, 20 Marie-Curie, Ottawa, Ontario, Canada; Institute of Molecular and Cell Biology, SINGAPORE

## Abstract

The evolution of the tetrapod limb involved an expansion and elaboration of the endoskeletal elements, while the fish fin rays were lost. Loss of fin-specific genes, and regulatory changes in key appendicular patterning genes have been identified as mechanisms of limb evolution, however their contributions to cellular organization and tissue differences between fins and limbs remains poorly understood. During early larval fin development, *hoxa13a/hoxd13a*-expressing fin fold mesenchyme migrate through the median and pectoral fin along actinotrichia fibrils, non-calcified skeletal elements crucial for supporting the fin fold. Fin fold mesenchyme migration defects have previously been proposed as a mechanism of fin dermal bone loss during tetrapod evolution as it has been shown they contribute directly to the fin ray osteoblast population. Using the nitroreductase/metronidazole system, we genetically ablated a subset of *hoxa13a/hoxd13a*-expressing fin fold mesenchyme to assess its contributions to fin development. Following the ablation of fin fold mesenchyme in larvae, the actinotrichia are unable to remain rigid and the median and pectoral fin folds collapse, resulting in a reduced fin fold size. The remaining cells following ablation are unable to migrate and show decreased *actinodin1* mesenchymal reporter activity. Actinodin proteins are crucial structural component of the actinotrichia. Additionally, we show a decrease in *hoxa13a*, *hoxd13a*, *fgf10a* and altered *shha*, and *ptch2* expression during larval fin development. A continuous treatment of metronidazole leads to fin ray defects at 30dpf. Fewer rays are present compared to stage-matched control larvae, and these rays are shorter and less defined. These results suggest the targeted *hoxa13a/hoxd13a*-expressing mesenchyme contribute to their own successful migration through their contributions to actinotrichia. Furthermore, due to their fate as fin ray osteoblasts, we propose their initial ablation, and subsequent disorganization produces truncated fin dermal bone elements during late larval stages.

## Introduction

The evolution of limbs was a hallmark in vertebrate innovation. No longer restricted to aquatic environments, tetrapods rapidly radiated and conquered their new terrestrial niches [1–3]. Fore- and hindlimbs evolved from the pectoral and pelvic fins, respectively, of lobe-finned sarcopterygian fish [4–6]. The fossil record has yielded useful transitional tetrapod species to investigate changes in bone morphology crucial for the evolution of the limb from the sarcopterygian fish fin. Expansion and elaboration of the appendicular endochondral bone resulted in the three distinct limb regions common to all tetrapods: the stylopod, zeugopod, and the autopod [1, 7, 8]. Simultaneously, the fin rays, present in all extant fish were gradually reduced before being completely lost from the tetrapod limb [1, 7]. While gene regulation differences are being identified as mechanisms of limb evolution, more information is required to link the contributions of these regulatory differences to changes at the level of cellular organization and tissue patterning during this process.

By examining early fin and limb development we can identify diverging developmental or molecular processes that may have contributed to the expansion and reduction of appendicular endochondral and dermal bone, respectively. Early fin and limb outgrowth is initiated and maintained by an FGF feedback loop between the apical ectodermal ridge (AER) and the underlying mesenchyme [9–14]. During tetrapod limb development, the AER is maintained relatively longer (E12.5 in the mouse forelimb) than in pectoral fin development resulting in sustained signalling FGF signalling [15–16]. The contributions of long-term AER signalling on endochondral bone evolution have been previously proposed in Thorogood’s Clock model [13, 17]. During zebrafish pectoral fin development, the AER folds to form the apical fin fold as early as 36hpf [10, 13]. Despite this morphological change, there is evidence suggesting components of AER-FGF signalling are maintained in fish pectoral fins despite the transition into apical fin fold [14, 18]. The fin fold is supported by two rows of fibrils known as actinotrichia, the first fin exoskeletal elements formed. Actinotrichia are made of collagen and Actinodin [19–21]. The *actinodin* gene family (*actinodin 1–4*) (ZDB-GENE-030131-9105, ZDB-GENE-041105-2, ZDB-GENE-040724-185, ZDB-GENE-081022-5) which codes for structural proteins crucial for actinotrichia formation has been lost from the tetrapod genome during the fin-to-limb transition [22]. Beginning shortly after the onset of *actinodin* expression, actinotrichia fibrils form and support the pectoral fin fold as it extends distally [10, 19–21]. At this stage *actinodin* genes are expressed in the ectoderm at the border of the presumptive endochondral disk and fin fold [22–23]. At around 52hpf, *actinodin* expression begins in a second population of cells, referred here as fin fold mesenchyme, which migrate distally through the fin fold using the pre-existing actinotrichia as a scaffold [23–24]. This secondary activation of *actinodins* in the mesenchyme is proposed to contribute to the thickness and length of the pre-existing actinotrichia [19, 23–24]. Actinotrichia fibrils are also present in the median fin fold, which extends along the midline of the embryo from the 8^th^ somite to the end of the trunk of the embryo, with *actinodin* expression starting at 24hpf [22, 25–26]). In tetrapod limb development, no actinotrichia or fold forms. In the adult pectoral fin, the endochondral disc serves as a template for the proximal radial bones and novel cartilagenous condensations at the distal edge of the disc will ossify to become the distal radials [10]. The distal radials are linked to the dermal bone, calcified fin rays (lepidotrichia), which form via intramembranous ossification from fin fold mesenchyme [10]. The median fin fold will become the 3 unpaired fins of the adult zebrafish: dorsal, caudal and anal fins [25–26].

Early fin and limb patterning is regulated by the 5’*HoxA/D* (9–13) genes [27–29]. Two distinct phases of *5’HoxA/D* expression are responsible for proximal and distal appendicular patterning, respectively [30–31]. Each phase is activated by regulatory sequences, called regulatory landscapes, found either 5’ (Global control region) or 3’ (Early limb control region) to the *HoxA* and *HoxD* gene clusters [28]. Late phase *5’HoxA/D* expression is activated in distal limb mesenchyme in tetrapods, whereas expression occurs in the distal cells of the endochondral disc, and the fin fold mesenchyme during fish fin development [30, 32–33]. 5’*Hox* regulatory data from the spotted gar and mouse, respectively, highlight a deep homology between distal fin and limb mesenchyme [34–36]. In zebrafish, *hoxa13*-expressing mesenchymal cells migrate distally through the larval fin fold and contribute to adult fin dermal bone [33, 37]. Co-localization of *hoxa13* (ZDB-GENE-990415-5, ZDB-GENE-980526-365) and *and1*, known autopod and fin fold markers respectively also supports a role of *hoxa13* during fin development in basal fish species [18]. In mice, *Hoxa13*-expressing cells do not migrate and instead contribute to the endochondral bones of the autopod [38]. Several hypotheses propose fin fold mesenchyme migration defects may be a mechanism of fin dermal bone loss during limb evolution [22, 30, 33]. To that end, we set out to create fin fold mesenchyme defects in the zebrafish to assess the effects on larval fin development.

We utilized the nitroreductase/metronidazole (NTR/MTZ) system to specifically ablate a subset of *hoxa13a/hoxd13a*-expressing cells during fin development. Briefly, in the presence of the NTR enzyme, MTZ substrate is converted to a cytotoxic compound leading to the death of the NTR-expressing cells. Using regulatory elements specific for fin mesenchyme, we can drive NTR in transgenic fish and specifically ablate fin fold mesenchyme upon MTZ addition to the fish water, while not producing any bystander effects [39–40]. In order to ablate fin fold mesenchymal cells prior to and during migration within the median and pectoral fin folds, we utilized the previously characterized “*m-Inta11”* regulatory element [32]. This regulatory element initiates antisense transcription at the *Hoxa11* exon 1 locus in mice, leading the distal repression of *Hoxa11* (MGI:96172). Using ChIP analysis, HOXA13 (MGI:96173) and HOXD13 (MGI:96205) have previously been shown to bind to this enhancer element in mice, and only *Hoxa13* -/- *Hoxd13* -/- double mutant mice show no activation of this enhancer element (single mutant mice show reduced enhancer activation) [32]. Furthermore, transgenic reporter zebrafish lines: *Tg(m-Inta11-β-globin:eGFP)* show reporter activation within the *hoxa13a* expression domain in the median and pectoral fins, further confirming a regulatory link between HOXA13 and the *m-Inta11* enhancer [32].

In the present study, we show that the ablation of fin fold mesenchymal cells during median and pectoral fin development results in fin fold collapse and actinotrichia defects. In addition, we observed endoskeletal disc reduction in the pectoral fin bud, as well as shifts in expression profiles of several key fin patterning genes. This suggests fin fold mesenchyme is crucial for the maintenance of actinotrichia and the fin fold during larval development. In addition, sustained metronidazole exposure for 30 days leads to fin ray defects in the pectoral fins at late larval stages, accompanied with a premature calcification in the proximal regions of the anterior-most fin rays compared to stage-matched controls. We propose that fin fold mesenchyme ablation, compounded by the lack of larval fin fold and actinotrichia maintenance, and distal reduction in *hoxa13a/hoxd13a* expression results in pectoral fin dermal bone defects.

## Results

### Subset of *hoxa13a/hoxd13a*-expressing cells are specifically ablated in *Tg(Inta11:NTR)*larvae following metronidazole treatment

**Fig 1:**
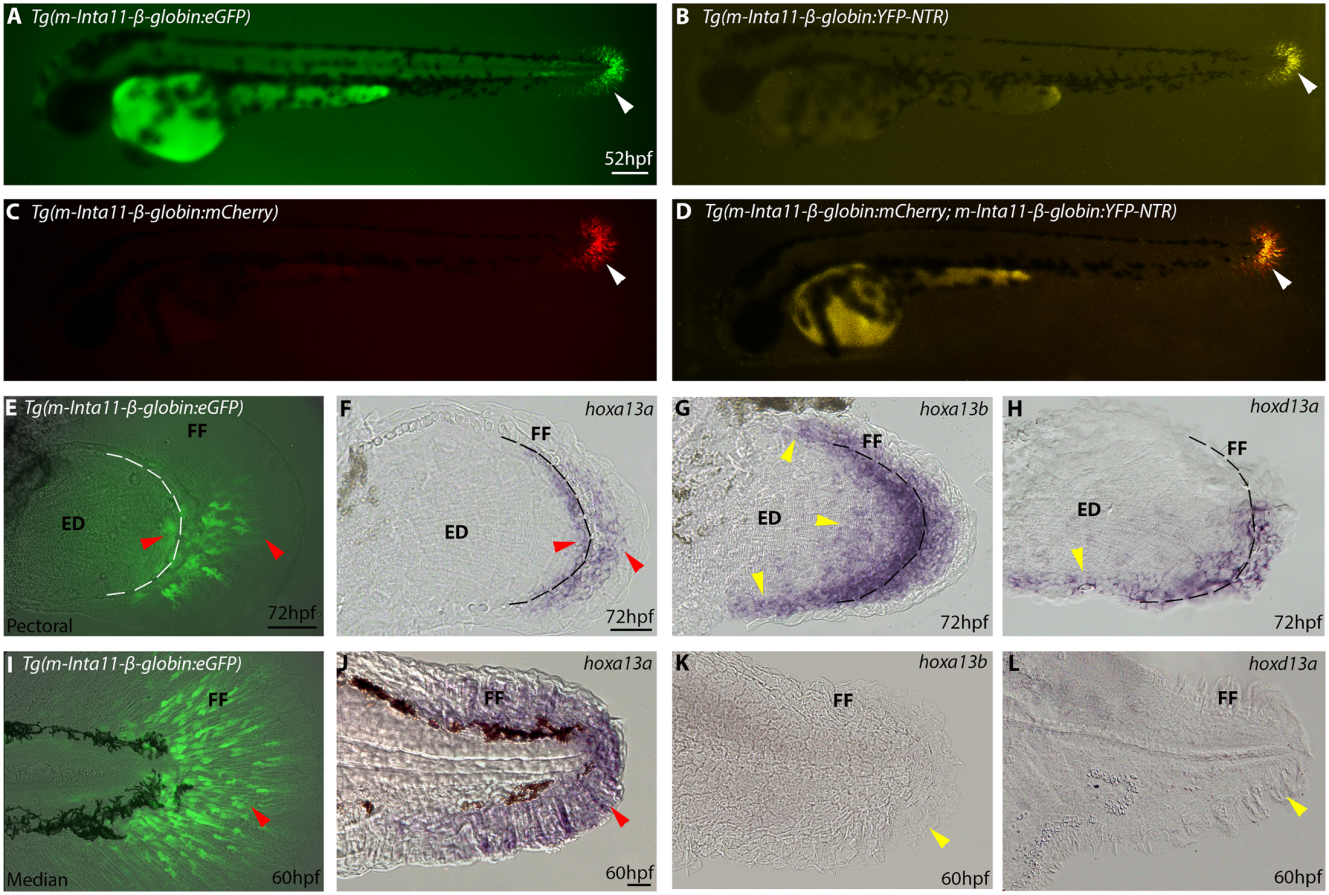
Nitroreductase (NTR) in *Tg(Inta11:NTR)* is expressed in subset of *hoxa13a/hoxd13a*-expressing mesenchyme of pectoral fin fold and *hoxa13a*-only expressing mesenchyme of the median fin fold. **(A-D)** Whole mount view of transgenic lines using the “*m-Inta11-β-globin*” regulatory elements at 52hpf. **(E-H)** Pectoral fin dissections showing reporter, *hoxa13a*, *hoxa13b*, and *hoxd13a* expression in the fin fold mesenchyme at 72hpf. **(I-L)** Median fin dissections showing reporter, *hoxa13a*, *hoxa13b*, and *hoxd13a* expression in the fin fold mesenchyme at 60hpf. At 52hpf, transgene (*eGFP*, *mCherry*, *YFP-NTR*) expression is visible in the migrating mesenchyme of the median fin fold using the “*m-Inta11-β-globin*” regulatory elements (white arrow) (A-D). Double transgenic fish *Tg(m-Inta11-β-globin:mCherry; m-Inta11-β-globin:YFP-NTR)* show colocalization of mCherry and YFP expressing cells in the median fin fold (D). Reporter expression is present in the migrating mesenchyme within the pectoral fin fold, as well as cells located at the distal edge of the endoskeletal disc (red arrow) (E), recapitulating a subset of *hoxa13a/hoxd13a*-expressing cells (F). *hoxa13b*, and *hoxd13a* expression extends proximally within the endoskeletal disk and this region does not correlate with reporter expression (yellow arrows) (G, H). Dotted line represents limit between fin fold and endoskeletal disc (F-H). Reporter expression is present in the migrating mesenchyme within the median fin fold (red arrow) (I), recapitulating endogenous *hoxa13a* expression (J). No *hoxa13b* or *hoxd13a* expression is visible in the median fin at 60hpf (yellow arrows) (K, L). Brightfield (F-H, J-L), fluorescence (A-D), and brightfield/fluorescence merged images (E, I K). ED, Endoskeletal disc; FF, Fin fold. Scale bars: 200μm in A-D; 50μm in E-G, I-L; 30μm in H.

To ablate fin fold mesenchyme in the median and pectoral fin fold, we utilized the previously characterized “*m-Inta11”* regulatory element [32] inserted upstream of the human *β-globin* minimal promoter to generate the *Tg(m-Inta11-β-globin:YFP-NTR)* transgenic line, thereafter named *Tg(Inta11:NTR*). We previously showed that the *m-Inta11-β-globin* regulatory elements drive transgene reporter expression in the *hoxa13a*-expressing fin fold mesenchyme of the pectoral fin buds (Fig 1E and [32]). Here we show that the transgenic reporter zebrafish line *Tg(m-Inta11-β-globin:eGFP)* also expresses the reporter gene in *hoxa13a*-expressing fin fold mesenchyme of the median fin (Fig 1A and 1I). To ensure NTR expression is consistent with the previously described *Tg(m-Inta11-β-globin:eGFP)* transgenic line, we produced another transgenic line: *Tg(m-Inta11-β-globin:mCherry)* to show co-localization of YFP and mCherry within the fin fold mesenchyme (Fig 1C and 1D). Despite evidence that both HOXA13 and HOXD13 bind to this enhancer in mice, we propose Hoxa13a as the main contributor to *m-Inta11* activation in the median fin fold. Neither *hoxa13b*, nor *hoxd13a* (ZDB-GENE-990415-119) are expressed in the median fin fold at 60hpf (Fig 1K and 1L), however we acknowledge Hoxa13b or Hoxd13a may still be contributing to enhancer activation due to protein persistence or a delay in reporter activity following binding. In the pectoral fin fold *hoxa13b* and *hoxd13a* expression extends more proximally in the endoskeletal disc regions of the pectoral fin at 72hpf, not consistent with *Tg(m-Inta11-β-globin:eGFP)* reporter expression nor *hoxa13a* expression (Fig 1E–1H). *Tg(m-Inta11-β-globin:eGFP)* larvae do show more eGFP-positive fin fold mesenchyme in the posterior half of the pectoral fin fold (Fig 1E), comparable to *hoxd13a* transcript localization (Fig 1H), suggesting Hoxd13a is likely contributing to enhancer activation in this region. Where therefore propose the *m-Inta11-β-globin* regulatory elements show activity in a subset of *hoxa13a/hoxd13a*-expressing cells in the pectoral fin, and *hoxa13a* only-expressing mesenchymal cells in the median fin fold. The *Tg(Inta11:NTR)* transgenic line recapitulates the previously described *Tg(m-Inta11-β-globin:eGFP)* transgenic line (Fig 1B).

**Fig 2:**
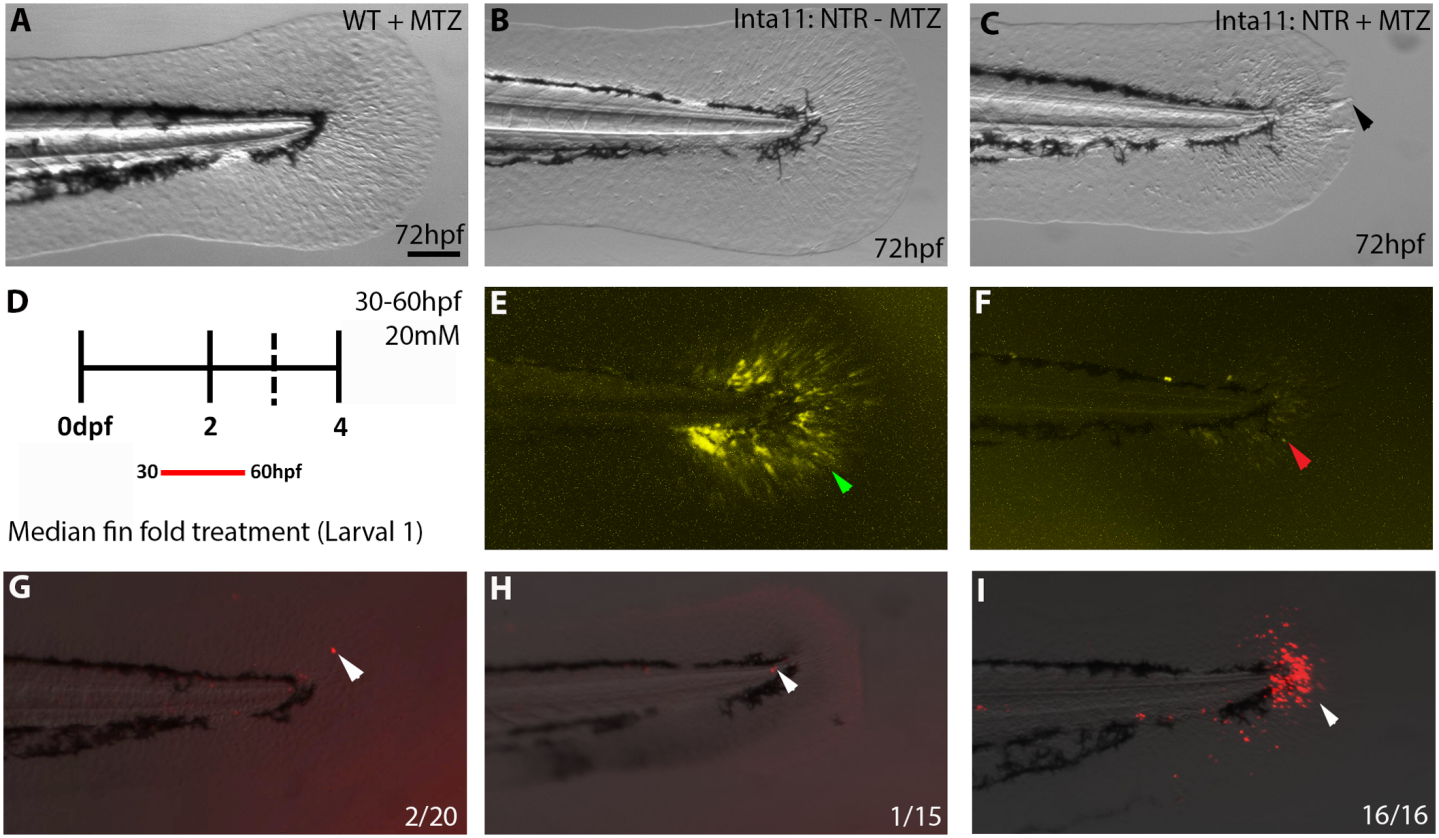
Subset of *hoxa13a/hoxd13a*-expressing cells specifically ablated in *Tg(Inta11:NTR)*fish following metronidazole treatment. **(A-C, E-I)** Median fin fold of 72hpf larvae from 3 treatment groups (2 control, 1 experimental), YFP expression levels and TUNEL assay are shown. **(D)** Schematic of “Larval 1” treatment, larvae are exposed from 30-60hpf. Median fin morphology unaffected in treatment control groups (WT + MTZ, Inta11: NTR—MTZ) (A, B) compared to Inta11: NTR + MTZ (C). Inat11: NTR + MTZ larvae show median fin fold collapse (black arrow) (C). YFP expression drastically reduced in Inta11: NTR + MTZ larvae (red arrow) (F), when compared to Inta11: NTR—MTZ (green arrow) (E). A small percentage of treated control larvae (10% and 6.66%) display single TUNEL-positive cells in the median fin fold (white arrow) (G, H). All treated Inta11: NTR + MTZ larvae (n = 16) show TUNEL-positive cells in the median fin fold (white arrow) (I). Brightfield (A—C), fluorescence (E, F), and brightfield/fluorescence merged images (G-I). Scale bars: 100μm in A-C, E-I.

Treatments of larvae with metronidazole were initiated at the onset of *Inta11:NTR* transgene expression, designed to encompass the peak of *hoxa13a/hoxd13a*-expressing cell migration in each fin. For median fin analysis “larval 1” treatment is performed spanning from 30-60hpf, and for pectoral fin analysis “larval 2” treatment is performed spanning from 52-72hpf (S1 Fig). Each experiment consists of one experimental group: *Tg(Inta11:NTR)* larvae treated with MTZ (Inta11: NTR + MTZ) and two control groups: WT larvae treated with MTZ (WT + MTZ), and *Tg(Inta11:NTR)* larvae treated with DMSO alone (Inta11: NTR—MTZ)(Fig 2A–2C). To ensure our system can specifically and consistently ablate fin fold mesenchyme in larval zebrafish we examined YFP expression in the median fin fold. Following “Larval 1” metronidazole (MTZ)-treatment, (Fig 2D and S1 Fig) *Tg(Inta11*: *NTR)* larvae show a drastic decrease in YFP expression in the median fin fold at 72hpf (Fig 2C and 2F), compared to untreated transgenic larvae (Fig 2B and 2E). The median fin fold of WT (non-transgenic) larvae that received MTZ developed normally (Fig 2A). To confirm the loss of YFP expression is indicative of cell death, a TUNEL assay was performed. Following “Larval 1” treatment (Fig 2D and S1 Fig), all MTZ-treated *Tg(Inta11*: *NTR)* larvae showed TUNEL-positive cells in the median fin fold at 72hpf (Fig 2I) (n = 16/16). Control fish groups present single TUNEL-positive cells in 1–2 larvae at 72hpf (Fig 2G and 2H). Consistently, *hoxa13a*, and *hoxd13a* expression is reduced in both the median and pectoral fins following MTZ-mediated ablation (see below). Altogether, these results confirm that treatment with metronidazole of the *Tg(Inta11:NTR)* transgenic line specifically ablates a subset of *hoxa13a/hoxd13a*-expressing fin fold mesenchyme.

### Morphological and migratory defects of the pectoral and median fin fold mesenchyme in *Tg(Inta11:NTR)* larvae following metronidazole treatment

**Fig 3:**
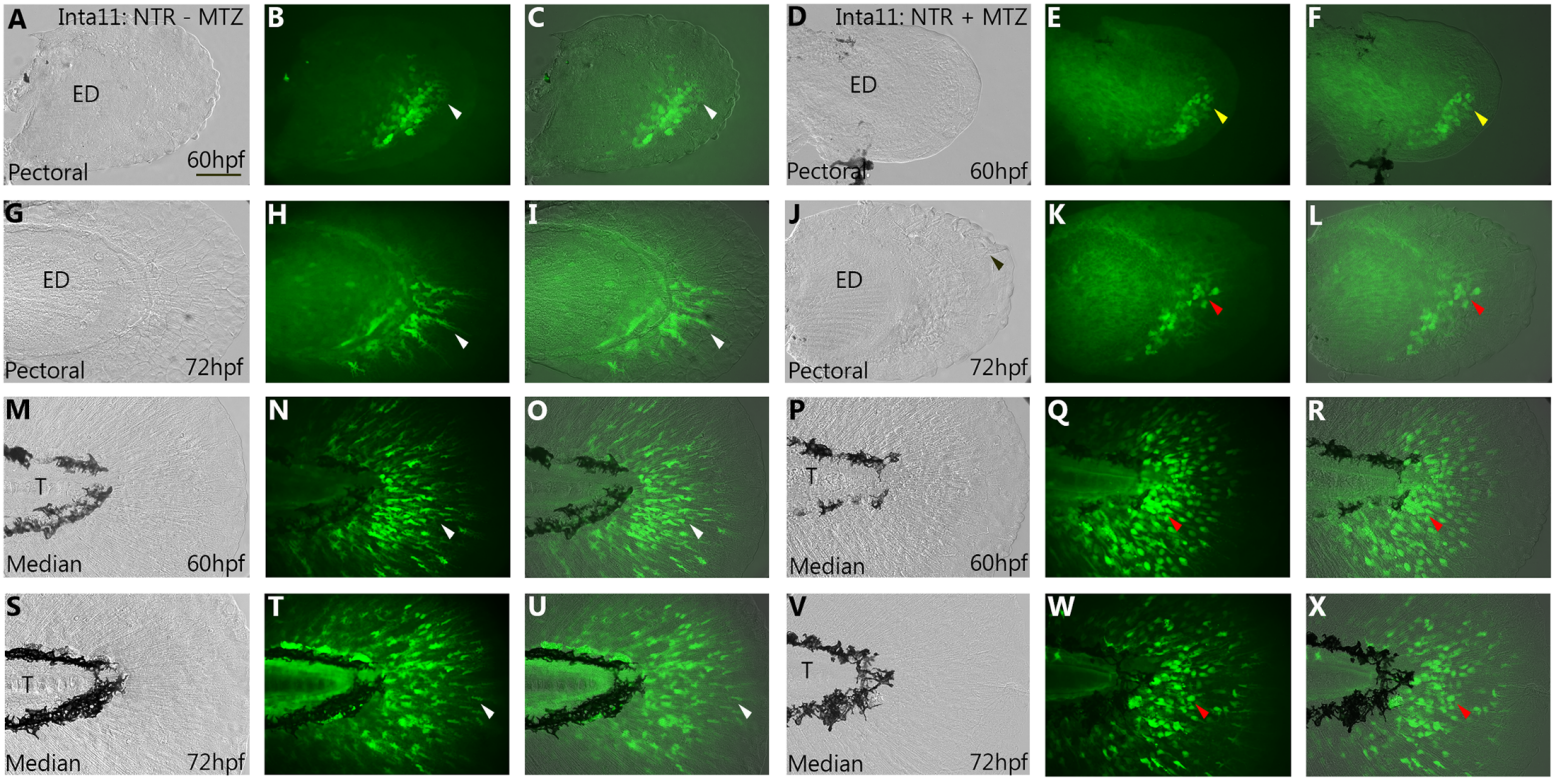
Morphological and migratory defects of the pectoral and median fin fold mesenchyme in *Tg(Inta11:NTR)* larvae following metronidazole treatment. **(A-J)** Pectoral and **(M-W)** median fin of 60, and 72hpf Inta11: NTR + MTZ and Inta11: NTR—MTZ larvae outcrossed with *Tg(Inta11-β-globin:eGFP)* transgenic larvae. At 60hpf, Inta11: NTR—MTZ show the beginning of fin fold migration in the pectoral fin (white arrow) (A-C). Migration is absent/delayed in the pectoral fin of the Inta11: NTR + MTZ group (yellow arrow) (D-F). At 72hpf, Inta11: NTR + MTZ larvae display reduced fin fold mesenchyme migration in the pectoral fin (J-L) compared to the control (G-I). Fin fold mesenchyme are less elongated/branched and are clustered close to the endoskeletal disk (red arrow) (J-L), compared to control pectoral fins (white arrow) (G-I). At 60, and 72hpf median fin fold mesenchyme of Inta11: NTR + MTZ larvae cluster next to the trunk, and are more round and less elongated/branched (red arrow) (P-R, V-X), compared to control larvae (white arrow) (M-O, S-U). Brightfield (A, D, G, J, M, P, S, V), fluorescence (B, E, H, K, N, Q, T, W), and brightfield/fluorescence merged images (C, F, I, L, O, R, U, X). ED, Endoskeletal disc; T, Trunk. Scale bars: 50μm in A-X.

To facilitate visualization of the fin fold mesenchyme defects following metronidazole treatments, *Tg(Inta11*: *NTR)* zebrafish were outcrossed to *Tg(m-Inta11-β-globin:eGFP)*, resulting in decreased ablation efficiency and higher number of surviving fin fold mesenchymal cells (Figs 1F and 3A–3X). For pectoral and median fin analysis, larvae were treated according to “larval 2”, and “larval 1” treatments, respectively (S1 Fig). At 60hpf, mesenchymal cells begin to migrate in the fin fold of the pectoral fin of untreated *Tg(Inta11*: *NTR)* larvae (Fig 3A–3C). In contrast, no actively migrating cells are present in the pectoral fins of treated *Tg(Inta11*: *NTR)* larvae (Fig 3D–3F), indicating a delay in migration. At 72hpf, untreated *Tg(Inta11:NTR)* larvae have actively migrating fin fold mesenchyme in proximal-posterior region of the pectoral fin fold, with several cells extending distally (Fig 3G–3I). MTZ-treated larvae show reduced fin fold mesenchyme migration and cells are less elongated (more rounded) in shape (Fig 3J–3L). Similar observations can be made for the median fin fold. At 60 and 72hpf, untreated larvae show actively-migrating fin fold mesenchyme through the median fin (Fig 3M–3O and 3S–3U). Cells are elongated and branched in shape. In contrast, treated larvae display clusters of mesenchymal cells in the fin fold surrounding the trunk region that have failed to initiate migration (Fig 3P–3R and 3V–3X). Furthermore, cells are rounded and unbranched in shape. These results show that following metronidazole treatment, mesenchymal cells in *Tg(Inta11*: *NTR)* larvae transition from an elongated, branched morphology to a rounded, unbranched morphology (a characteristic of dying cells) and subsequently fail to migrate properly.

### Actinotrichia defects and fin fold collapse in pectoral and median fins of *Tg(Inta11:NTR)* following metronidazole treatment

**Fig 4:**
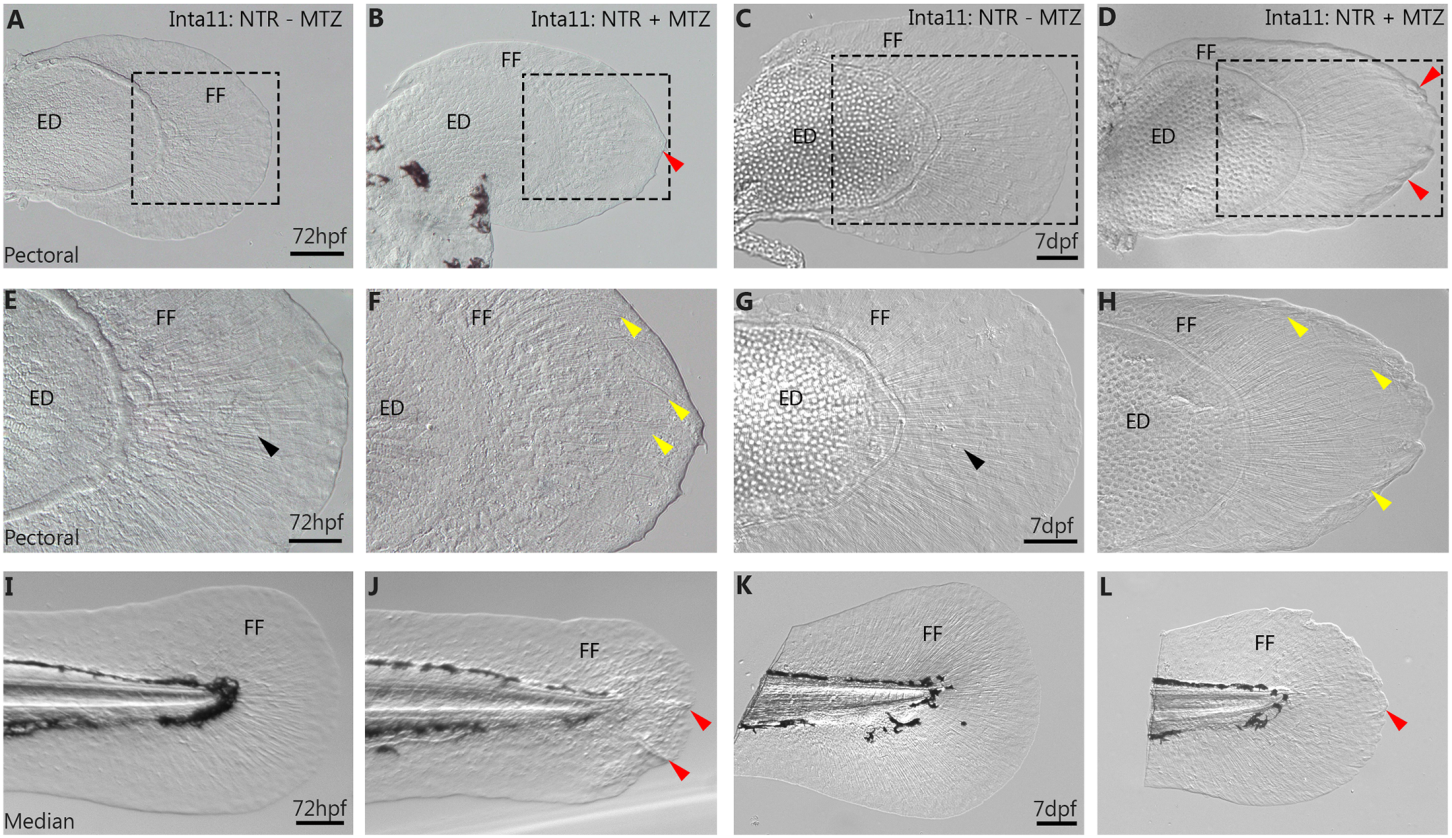
Fin fold collapse in 72hpf, 7dpf pectoral and median fins of *Tg(Inta11:NTR)* following metronidazole treatment. **(A-H)** Pectoral and **(I-L)** median fins of Inta11: NTR + MTZ and Inta11: NTR–MTZ control larvae at 72hpf and 7dpf. Inta11: NTR + MTZ larvae display pectoral fin fold collapse at 72hpf (B, F) and 7dpf (D, H), compared to Inta11: NTR—MTZ (A, C, E, G). Note the collapse of the fin fold (red arrows) (B, D). Panels E-H are magnifications of dotted box in panels A-D. Note the appearance of bending actinotrichia fibrils (yellow arrows) in Inta11: NTR + MTZ larvae (F, H) compared to straight actinotrichia (black arrows) in the Inta11: NTR—MTZ larvae (E, G). Inta11: NTR + MTZ larvae display major median fin fold defects at 72hpf (J) compared to Inta11: NTR—MTZ larvae (I). Note the collapse of the fin fold (red arrows) (J). By 7dpf, Inta11: NTR + MTZ larvae continue to show a reduction in median fin fold size compared to Inta11: NTR—MTZ larvae (K), however defects are ameliorated compared to Inta11: NTR + MTZ larvae at 72hpf (J, L). Note the minor folding of distal tip of the median fin (red arrow) (L). ED, Endoskeletal disc; FF, Fin fold. Scale bars: 100μm in A-D, F, H, I-L; 50μm in E, G.

**Fig 5:**
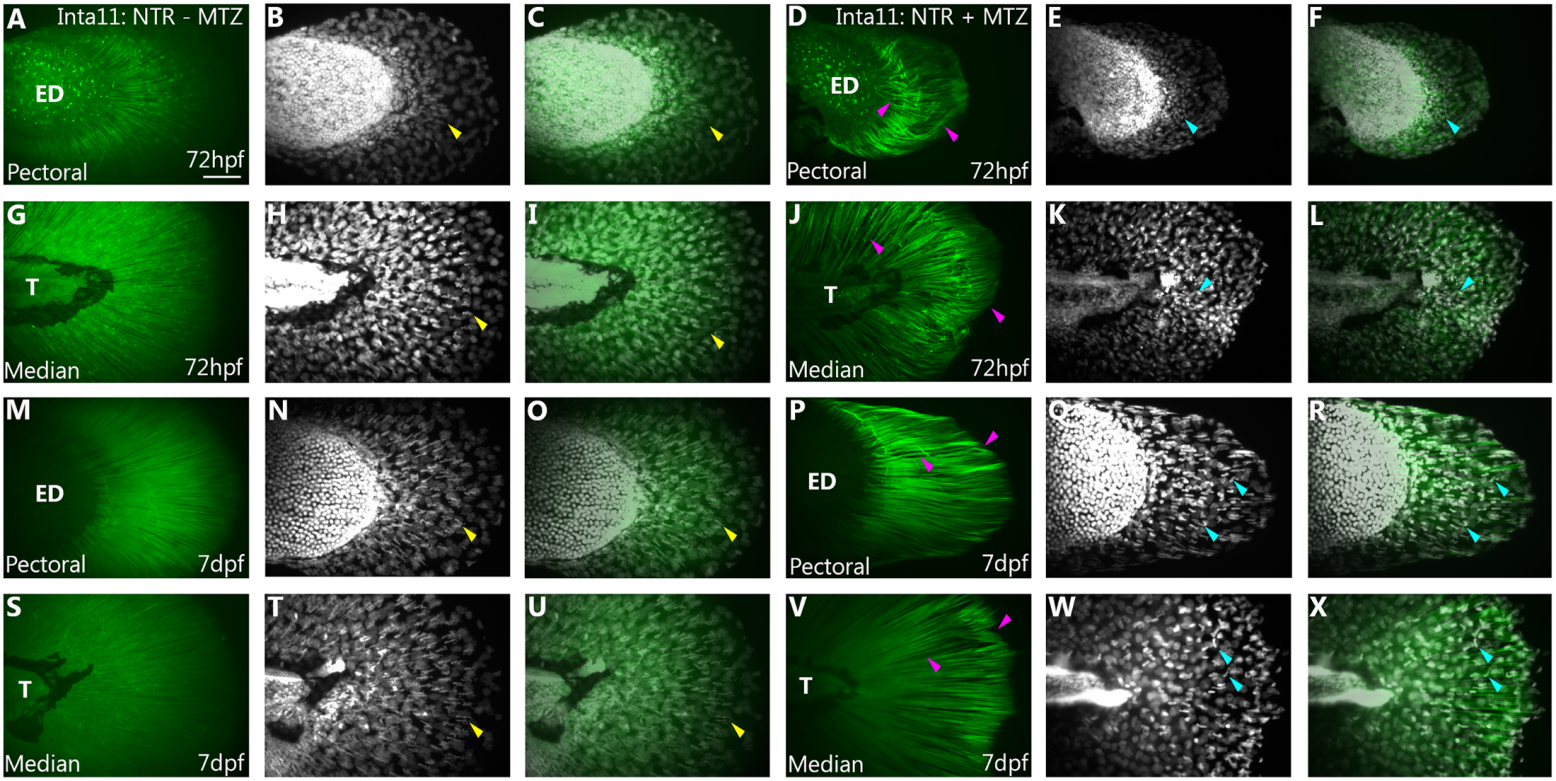
Actinotrichia defects in 72hpf, 7dpf pectoral and median fins of *Tg(Inta11:NTR)* following metronidazole treatment. Collagen II Immunostaining of **(A-F, M-R)** pectoral and **(G-L, S-X)** median fins of Inta11: NTR + MTZ and Inta11: NTR–MTZ control larvae at 72hpf and 7dpf. At 72hpf, and 7dpf untreated larvae show rigid, parallel actinotrichia throughout the pectoral and median fin fold (A, C, G, I, M, O, S, U), with DAPI staining revealing proper fin fold mesenchymal cell migration (Yellow arrow) (B, H, N, T). Note the fin fold mesenchyme elongate along the proximal distal axis, aligning with the actinotrichia (Yellow arrow) (B-C, H-I, N-O, T-U). At 72hpf, and 7dpf, actinotrichia of MTZ-treated larvae are unable to remain rigid and bend within the fin fold (Purple arrow) (D, F, J, L, O, R, V, X). This correlates with fin fold collapse. The actinotrichia are unable to remain parallel to one another, creating gaps within the fin fold (D, F, J, L, O, R, V, X). Note the apparent unbundling of Collagen II stained strands at 72hpf (Purple arrow) (D, J). At 72hpf, DAPI staining reveals fin fold mesenchyme cluster next to the pectoral fin endoskeletal disc and the trunk region proximal to the median fin fold (Teal arrow) (E, K), having failed to migrate correctly. At 7dpf, surviving fin fold mesenchyme fails to migrate correctly (Teal arrow) (Q, W). In the pectoral fin, migration is restricted to the central region of the fin fold (Teal arrow) (Q) and in both the pectoral and median fin, these cells display elongation along various different axes, correlating with actinotrichia defects (Teal arrow) (P-R, V-X). Collagen II staining (A, D, G, J, M, P, S, V), DAPI (B, E, H, K, N, Q, T, W) and merged (C, F, I, L, O, R, U, X) images are presented. ED, Endoskeletal disc, T, Trunk Scale bars: 50μm in A-X.

We propose that the observed decrease in median and pectoral fin fold size is due to fin fold collapse resulting from actinotrichia defects. At 72hpf, both the median and pectoral fins of MTZ-treated *Tg(Inta11*: *NTR)* larvae show signs of fin fold collapse (Fig 4B, 4F and 4J) when compared to untreated controls (Fig 4A, 4E and 4I). This was seen following “larval 1” or “larval 2” treatments. At closer magnification, fins of treated individuals show disorganized actinotrichia in the pectoral fin, with bending that is parallel to the fin fold collapse (Fig 4F). In order to observe actinotrichia structure in both the median and pectoral fin, we performed immunohistochemistry for Collagen type II, which has previously been shown to label actinotrichia during larval development [19] (Fig 5). At 72hpf, actinotrichia of MTZ-treated larvae are unable to remain rigid, and bend within the fin fold (Fig 5D–5F and 5J–5L). This correlates with fin fold collapse along the entire edge of the pectoral and median fin fold (Fig 5D–5F and 5J–5L). The actinotrichia do not remain parallel to one another, consistently revealing gaps between the fibrils. Furthermore, there is an apparent unbundling of CoIlagen II stained strands (Fig 5D and 5J). Fin fold migration defects can also be observed using DAPI staining, however cell mesenchyme morphology is not as evident. Fin fold mesenchyme nuclei mimic cell shape but the extent of cellular elongation is not visible, precluding the use of DAPI staining for cell displacement measurements. Fin fold mesenchyme of MTZ-treated larvae clustered near endoskeletal disk of the pectoral fin, and the trunk region anterior to the median fin (Fig 5E, 5F, 5K and 5L). Untreated larvae show straight, rigid actinotrichia throughout the median and pectoral fin fold, with visible fin fold mesenchyme migration (Fig 5A–5C and 5G–5I). Fin fold mesenchyme produce and secrete actinodin proteins, and we propose their ablation results in a failure to maintain the actinotrichia fibres and in the subsequent collapse of the fin fold. To maintain ablation effects until 7dpf, a secondary metronidazole treatment, “larval 3”, from 72hpf -7dpf, is required to suppress fin fold mesenchyme regeneration following “larval 1 & 2” treatments (S1 and S4 Figs). At 7dpf, MTZ-treated *Tg(Inta11:NTR)* larvae continue to show severe collapse of the fin fold and actinotrichia defects in the pectoral fin (Figs 4D, 4H and 5P–5R) compared to untreated larvae (Fig 5C, 5G and 5M–5O), following a combination of “larval 2”+ “larval 3” treatments (S1 Fig). The actinotrichia are still unable to remain parallel and bend at the distal tip of the fin fold. The most severe defects are at the anterior and posterior regions where the actinotrichia bend inward correlating with fin fold collapse along the anterior-posterior axis (Fig 5P). Untreated larvae show rigid parallel actinotrichia throughout the pectoral fin fold (Fig 5M–5O). DAPI staining reveals two-fold defects in surviving fin fold mesenchyme migration: migrating cells are now restricted centrally having converged inward from anterior-posterior fin fold collapse, and many cells show improper orientation that seems to correlate with the actinotrichia defects (Fig 5P–5R). Many cells appear to elongate along several different axes within the fin fold compared to untreated larvae that show cell elongation restricted to the proximal-distal axis (Fig 5Q, 5R, 5N and 5O). At 7dpf, MTZ-treated *Tg(Inta11:NTR)* larvae continue to show a reduction in size of the median fin fold compared to untreated control larvae, (Fig 4L and 4K) following “larval 1” and “larval 3” treatments (S1 Fig). Actinotrichia defects are also observed in the median fin of MTZ-treated *Tg(Inta11:NTR)* larvae (Figs 4J–4L and 5V–5X) compared to control larvae (Figs 4I, 4K and 5S–5U). Similar to observations in the pectoral fin, fin fold mesenchymal cells appear to elongate along different axes compared to untreated larvae (Fig 5W, 5X, 5T and 5U). Median fin fold defects are ameliorated in treated larvae at 7dpf, (Fig 4L) when compared to 72hpf median fins (Fig 4J). We believe median fins trend towards a complete reversal of the 72hpf phenotype (Fig 4J) due to incomplete median fin fold mesenchyme ablation. This aspect will be addressed in the discussion.

### In, Metronidazole-treated, *Tg(Inta11:NTR)* larvae show defects in median fin fold mesenchyme migration, a reduction in median and pectoral fin fold size and a reduction in endoskeletal disc size

**Fig 6:**
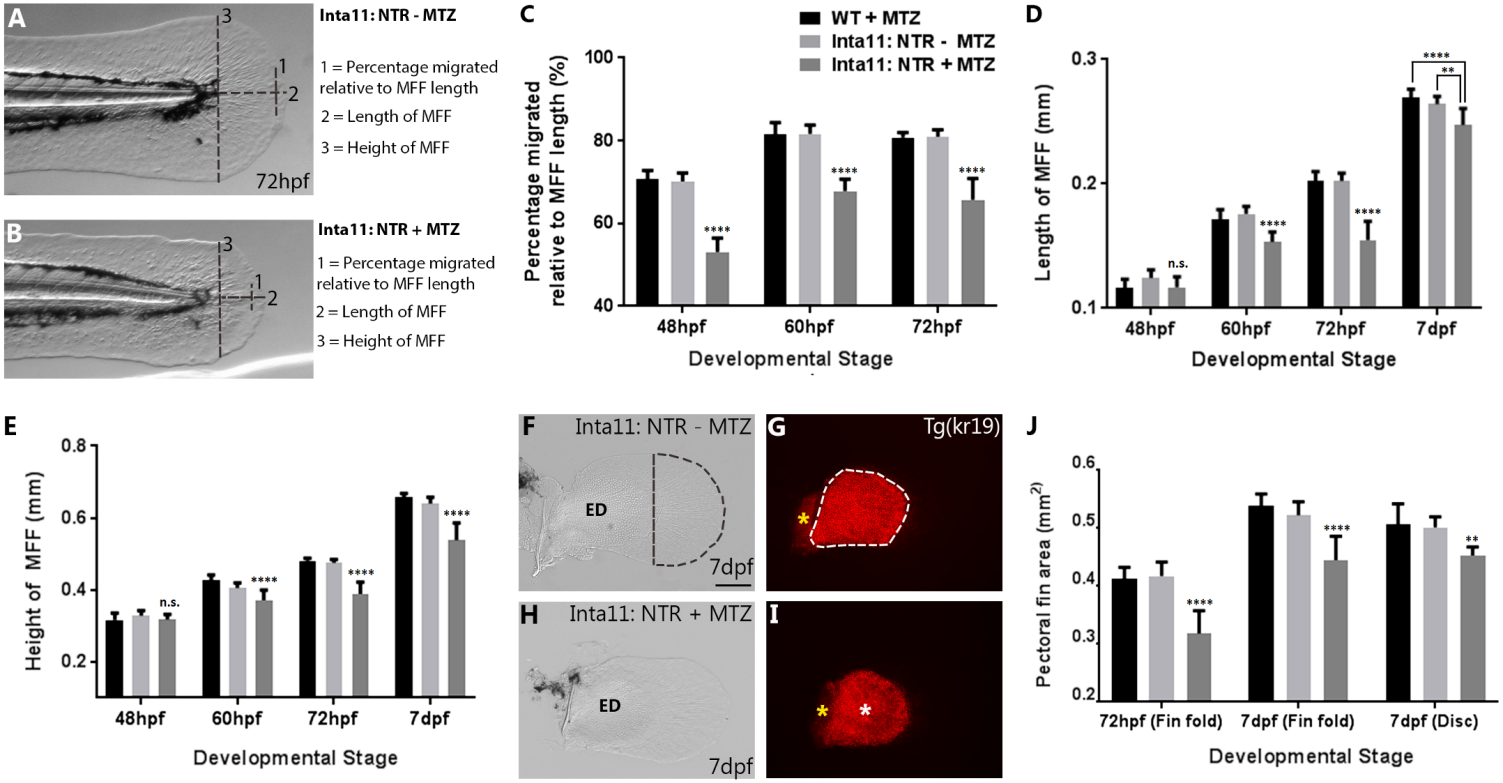
Metronidazole-treated *Tg(Inta11:NTR)* larvae show defects in median fin fold mesenchyme migration, a reduction in median and pectoral fin fold size and a reduction in endoskeletal disc size. **(A-B)** Schematic of median fin fold measurements. **(C-E, J)** Graphs displaying measurements of median fin mesenchyme displacement (%), median fin fold width (mm) and height (mm), and pectoral fin fold and endoskeletal disc area (mm^2^). **(F-G)** Inta11: NTR—MTZ and **(H-I)** Inta11: NTR + MTZ pectoral fin at 7dpf outcrossed with *Tg(kr19)* to highlight endoskeletal disc. Fin fold mesenchyme cell displacement is represented as a percentage displaced relative to the overall fin fold length (trunk to distal tip) (Measurement 1), length of median fin fold is measured from trunk to distal tip (measurement 2), and height of median fin fold is measured from dorsal to ventral tips at the trunk (measurement 3) (A-B). Inta11: NTR + MTZ larvae display a reduction in median fin fold mesenchyme cell displacement at 48, 60, and 72hpf compared to control larvae (C). Inta11: NTR + MTZ larvae show a reduction in median fin fold width and height at 60, 72hpf, and 7dpf compared to control larvae (D, E). No difference is observed for either measurement at 48hpf (D, E). Inta11: NTR + MTZ larvae show a decrease in pectoral fin fold area at 72hpf, and 7dpf, as well as a reduction in endoskeletal disc size at 7dpf (J). Example of Inta11: NTR—MTZ (F, G) and Inta11: NTR + MTZ (H, I) pectoral fin used for distal fin fold, endoskeletal disc measurements. Region used for measurement is indicated by dotted line (F, G). Note the decreased disc size in the Inta11: NTR + MTZ pectoral fin (white asterisks) (I). Scapulocoracoid not included in the disc area measurements (yellow asterisks) (G, I). All bar values are an average of 10 measurements (n = 10 fins) with standard deviation indicated, with the exception of endoskeletal disc size (J). Endoskeletal disc values are based on measurements of 5, 5, and 8 fins (n = 5 fins, n = 5 fins, n = 8 fins) for treatment controls and Inta11: NTR + MTZ larvae respectively. Standard one-way ANOVA was performed. Each mean was compared against both other means. Tukey’s correction was applied. No statistically relevant difference was ever detected between treatment controls (WT + MTZ, Inta11: NTR—MTZ). Inta11: NTR + MTZ P-values (asterisks) are representative of comparisons with both treatment controls, with the exception of median fin fold width at 7dpf, where unique P-values are indicated for comparisons with each control (D). Brightfield (A-B, F, H), fluorescence (G, I). P-values: ** P = 0.001>0.005, **** P = <0.0001. ED, Endoskeletal disc; MFF, Median fin fold. Scale bars: 100μm (F-I).

Following MTZ-mediated ablation of fin fold mesenchyme in *Tg(Inta11:NTR)*, we observed severe median fin fold collapse and reduced overall size (Fig 2C). In order to quantify these median fin fold defects, we measured fin fold length and height, as well as overall median fin fold mesenchyme cell displacement (Fig 6A–6E). Cell displacement was measured as a proportion of total median fin fold length (trunk to distal tip), and recorded as a percentage. All measurements are therefore relative to the size of the fin fold, eliminating any bias for a general impairment of fin fold growth. Following “Larval 1” or “Larval 3” treatments, median fins of all groups were examined at 48, 60, 72hpf and 7dpf. MTZ-treated *Tg(Inta11:NTR)* animals display significantly reduced median fin fold mesenchymal cell displacement from 48 to 72hpf compared to both control groups (Fig 6C). At 48hpf, the median fin fold length and height of MTZ-treated *Tg(Inta11:NTR)* larvae display no difference compared to controls (Fig 6D and 6E). However significant reductions are observed starting at 60hpf and the defects are maintained through 7dpf (Fig 6D and 6E). To ensure the effects were not limited to the median fin, we measured the area of the distal pectoral fin fold and endoskeletal disc (Fig 6F–6I). Following “Larval 2” or “larval 3” treatments, the distal pectoral fin fold area is significantly reduced MTZ-treated *Tg(Inta11:NTR)* larvae at 72hpf compared to both groups of control larvae and the defect is maintained until 7dpf (Fig 6J). To measure the endoskeletal disc area, *Tg(Inta11:NTR)* fish were outcrossed with *Tg(Kr19)* transgenic fish (Fig 6G and 6I), where KillerRed is expressed in the endoskeletal disk, among other regions (Teh et al. 2010). At 7dpf, MTZ-treated *Tg(Inta11:NTR)* larvae show a decrease in endoskeletal disc size compared to control larvae (Fig 6J). These results show that following metronidazole treatment, cells targeted for ablation in *Tg(Inta11*: *NTR)* larvae show significant defects in their migration. MTZ-treated *Tg(Inta11:NTR)* fish also show a reduction in the size of the median and pectoral fin folds, as well as of the endoskeletal disc.

### *A*ltered gene expression profiles in the median and pectoral fin of *Tg(Inta11:NTR)* larvae following metronidazole treatment

**Fig 7:**
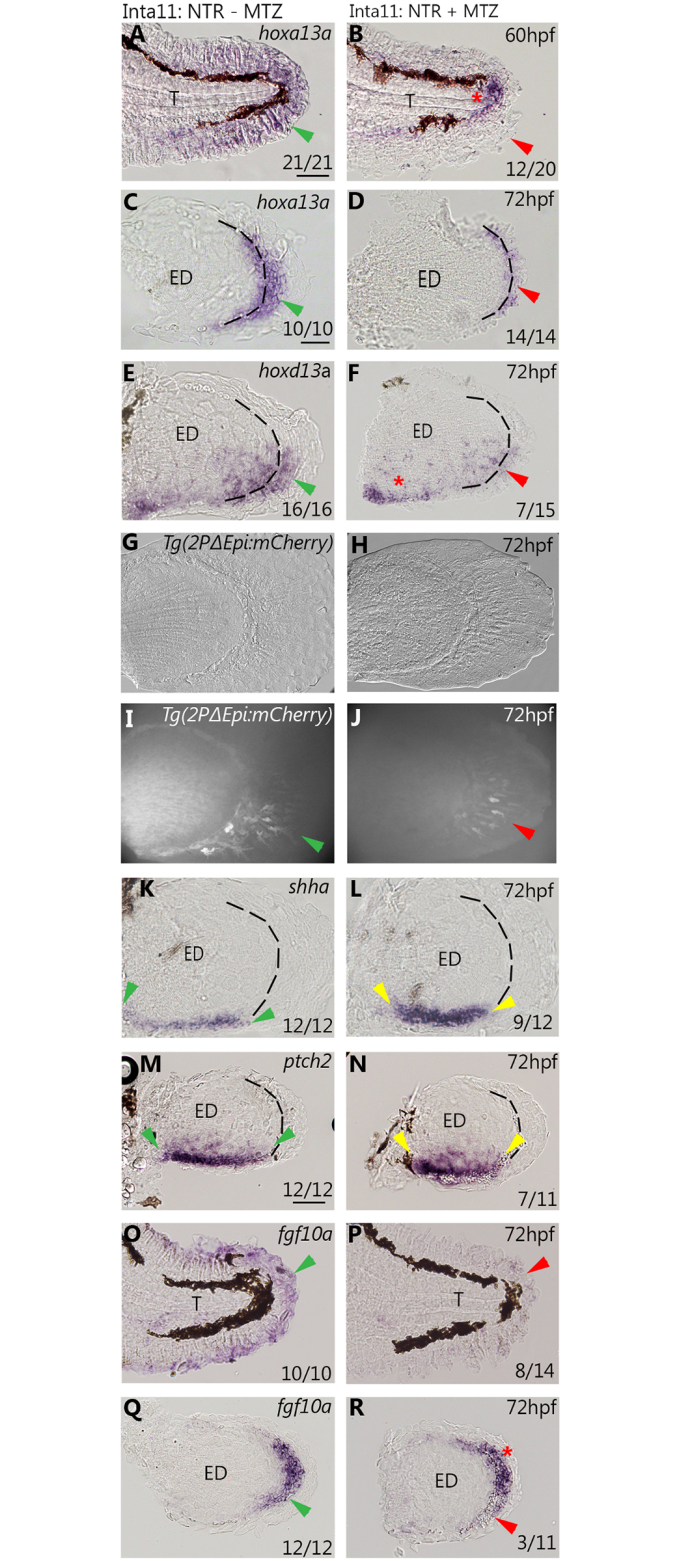
*A*ltered gene expression profiles in the median and pectoral fin of *Tg(Inta11:NTR)* larvae following metronidazole treatment. **(A-N)** *in situ hybridization* and *and1* reporter data showing gene expression profiles in the median and pectoral fin at 60, and 72hpf in Inta11: NTR—MTZ and Inta11: NTR—MTZ larvae. Inta11: NTR—MTZ are present in the left panels (A, C, E, G, I, K, M, O, Q) and Inta11: NTR + MTZ are present in the right panels (B, D, F, H, J, L, N, P, R). Inta11: NTR + MTZ show a decrease in distal *hoxa13a* expression (red arrow) in the median fin at 60hpf (B), and in the pectoral fin at 72hpf (D) compared to Inta11: NTR—MTZ (green arrow) (A, C). Note unaltered *hoxa13a* expression in the trunk region of Inta11: NTR + MTZ (red asterisks) (B). Inta11: NTR + MTZ show a decrease in distal *hoxd13a* expression (red arrow) in the pectoral fin at 72hpf (F) compared to Inta11: NTR—MTZ larvae (green arrow) (E). Note unaltered *hoxd13a* expression in the proximal disc region of Inta11: NTR + MTZ larvae (red asterisks) (F). Inta11: NTR + MTZ double transgenic larvae show decreased *and1* reporter activity (red arrow) (J) in the pectoral fin compared to Inta11: NTR—MTZ double transgenic larvae (Red arrow) (I) at 72hpf. Brightfield (G-H) and fluorescent (I-J) images are included. Inta11: NTR + MTZ larvae show an increased anterior-posterior, and decreased proximal–distal expression domain of both *shha* and its receptor *ptch2* in the pectoral fin at 72hpf (yellow arrows) (L, N) compared to Inta11: NTR—MTZ larvae (green arrow) (K, M). Inta11: NTR + MTZ show a decrease in distal/distal posterior *fgf10a* expression at 72hpf, in the median and pectoral fin respectively (red arrows) (P, R) compared to Inta11: NTR—MTZ larvae (green arrows) (O, Q). Note unaltered expression of *fgf10a* in the anterior pectoral fin mesenchyme of Inta11: NTR + MTZ larvae (red asterisks) (N). Dotted lines indicate fin fold and disk boundary (C-F, K-N, Q-R)). Probe or reporter line is indicated in the top right corner of each panel in the left column, age is indicated in the top right corner of each panel in the right column (A-R). Number of larvae displaying gene expression pattern, for *in situ* hybridization data, are indicated in the bottom right corner of each panel (A-F, K-R). WT-MTZ+DMSO images are contained in S1 Fig, and show similar expression profiles to Inta11: NTR—MTZ larvae (A, C, E, K, M, O, Q). ED, Endoskeletal disc; T, Trunk. Scale bars: 100μm in A, B, K, L; 50μm in I, J, M, N; 30μm in C-H.

In order to observe the consequences of fin fold mesenchyme ablation on gene expression and interpret the subsequent morphological defects, we performed whole mount *in situ* hybridization for several genes which play important roles in fin (and limb) development (Fig 7 and S2 Fig). As a subset of *hoxa13a/hoxd13a*-expressing cells are targeted for ablation, we first confirmed lower distal transcript levels of *hoxa13a* in the median and pectoral fins of MTZ-treated *Tg(Inta11:NTR)* at 60- and 72hpf (Fig 7B and 7D) compared to control larvae (Fig 7A and 7C and S2 Fig) and decreased *hoxd13a* expression in the distal pectoral fin fold and disc at 72hpf (Fig 7F) compared to control larvae (Fig 7E and S2 Fig). There is no change in *hoxd13a* expression in the proximal disc regions (Fig 7F). No visible change in expression is observed for *hoxa13b*, and *hoxa11b* (ZDB-GENE-990415-4) in the pectoral fins of MTZ-treated *Tg(Inta11*: *NTR)* larvae at 72hpf (S2 Fig). The domains of expression for *hoxa13b* and *hoxa11b* extend more proximally, outside the region targeted for ablation, and therefore the unaffected high levels of proximal transcripts may be masking the decreases in the distal domain. No change in *and1* ectodermal expression is visible by *in situ* hybridization (S2 Fig). Although a decrease in mesenchymal expression of *and1* is expected, it may be hidden by ectodermal expression of the same gene. The larval fin has *and1* expression in the fin fold ectoderm and mesenchyme. To show a decrease in *and1* in the mesenchyme, we outcrossed *Tg(Inta11:NTR)* fish with the *and1* mesenchymal reporter line: *Tg(2PΔEpi:mCherry)* [23]. The regulatory elements “2PΔEpi” contain multiple mesenchymal-specific enhancers, and the endogenous *and1* promoter [23]. Following metronidazole treatment, double transgenic larvae show fewer mCherry-positive cells in the pectoral 72hpf compared to untreated controls, indicating lower *and1* expression (Fig 7G–7J).

Expression of 5’*hox*A/D genes has been previously shown to be required for activation and maintenance of *Shh* expression during mouse limb development [41–42]. In MTZ-treated *Tg(Inta11:NTR)* larvae, both *shha* (ZDB-GENE-980526-166), and its receptor *ptch2* (ZDB-GENE-980526-44) display a smaller domain of expression along the proximal-distal axis of the posterior endoskeletal disc at 72hpf, as well as a slight anterior expansion (Fig 7L and 7N) when compared to control larvae (Fig 7K and 7M and S2 Fig). No difference in the expression of *hhip* (ZDB-GENE-030131-4827), coding for a shha antagonist, is observed in the pectoral fins of MTZ-treated *Tg(Inta11:NTR)* larvae compared to controls at 72hpf (S2 Fig). Altered SHH signalling via decreased 5’*Hox* transcripts has previously been associated with decreased endoskeletal disc proportions [43], and thus supports reduced endoskeletal disc size observed in MTZ-treated *Tg(Inta11:NTR)* larvae at 7dpf (Fig 6J).

AER-FGF signalling in tetrapod limb development relies on feedback loops with SHH and 5’HOX signalling [42, 44–46]. We therefore wished to determine if changes in expression of *fgf8a* (ZDB-GENE-990415-72), and *fgf10a* (ZDB-GENE-030715-1) are observed in MTZ-treated *Tg(Inta11*: *NTR)* larvae. At 72hpf, treated larvae show a clear decrease in *fgf10a* expression in the median fin fold compared to control larvae (Fig 7O and 7P and S2 Fig). Treated larvae show a slight decrease in *fgf10a* transcripts in the posterior fold mesenchyme of pectoral fins compared to controls (Fig 7Q and 7R and S2 Fig). Despite a decrease in *fgf10a* expression, no change in *fgf8a* expression is observed at 72hpf in pectoral fins of treated larvae compared to controls (S2 Fig). Decreased AER-FGF signalling has been associated with decreased endochondral bone elements, and therefore may also be causing the endoskeletal disc reduction observed in treated *Tg(Inta11:NTR)* larvae at 7dpf (Fig 6J) [11, 46–47]. The ablation of fin fold mesenchyme in *Tg(Inta11:NTR)* larvae following metronidazole treatment results in decreased *hoxa13a* and *hoxd13a* transcripts in the distal median and pectoral fins. We propose this decrease in overall 5’*hoxA/D* transcripts may result in a decrease in fgf signalling, possibly followed by a decrease in downstream shha signalling, and can account for the reduction in endoskeletal disc size observed in Inta11: NTR + MTZ larvae at 7dpf.

### Actinotrichia defects, fin fold collapse, and fin ray defects in pectoral fins of *Tg(Inta11:NTR)* fish during late larval stages following metronidazole treatment

**Fig 8:**
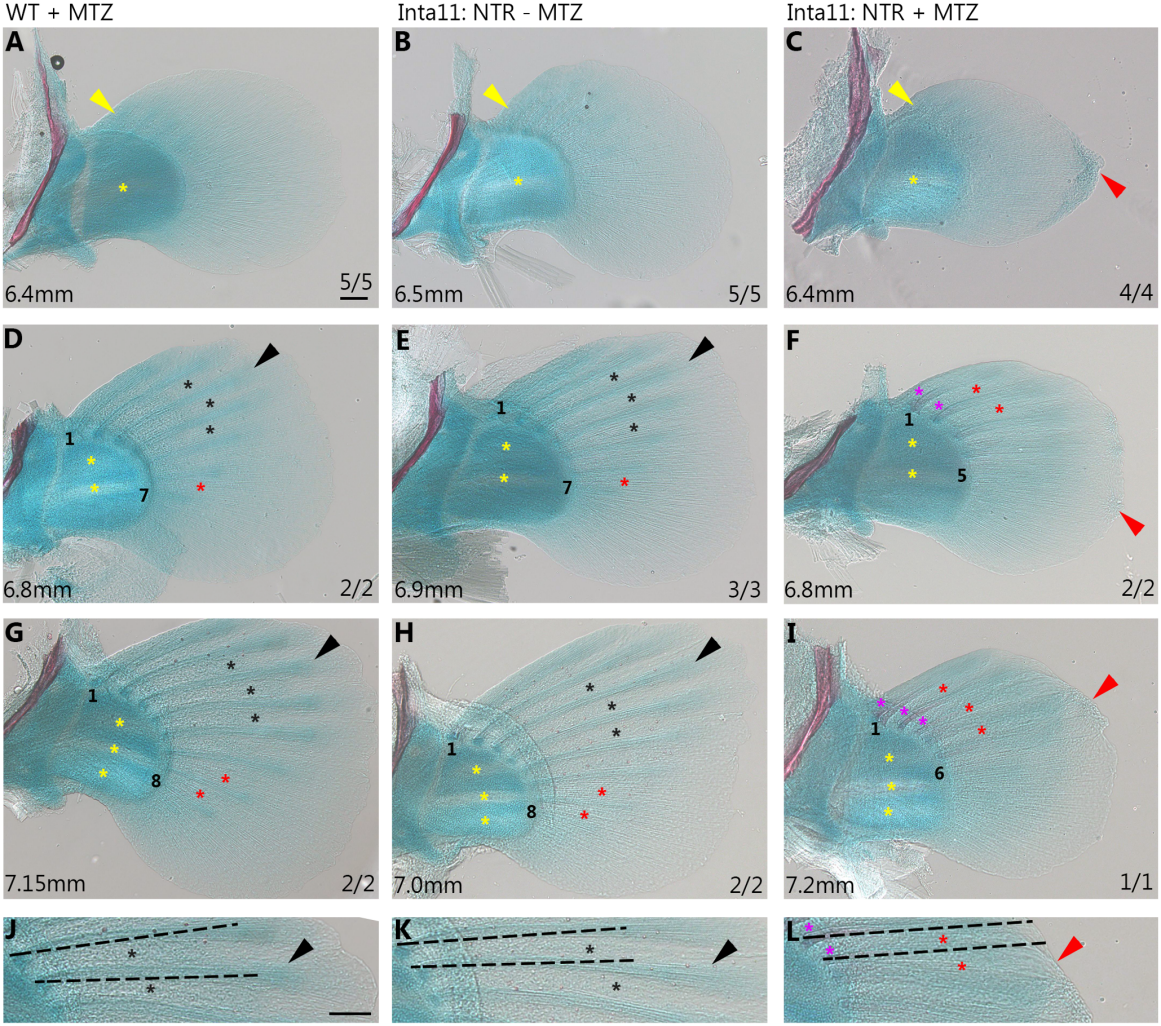
Actinotrichia defects, fin fold collapse, and fin ray defects in pectoral fins of *Tg(Inta11:NTR)* fish during late larval stages following metronidazole treatment. **(A-L)** Cartilage and bone stained pectoral fins of WT-MTZ+DMSO, Inta11:NTR—MTZ, and Inta11: NTR + MTZ larvae at three standard lengths (6.4, 6.8, and 7.2mm). At standard length 6.4mm, Inta11: NTR + MTZ larvae continue to show fin fold collapse (red arrow), and actinotrichia disorganization (C), compared to control larvae (A-B). Decomposition of disc cartilage matrix between presumptive proximal radials 2 and 3 has started (yellow asterisks) (A-C). Note, the initial stages of anterior-most lepidotrichia formation are not affected in the Inta11: NTR + MTZ larvae (C), compared to control larvae (A-B) (yellow arrows). At standard lengths 6.8mm and 7.2mm, Inta11: NTR + MTZ larvae have a reduced number of visible lepidotrichia compared to control larvae (D-I). Anterior-most and posterior-most visible rays have been numbered. Inta11: NTR + MTZ larvae continue to have minor collapse of the fin fold (red arrows) (F, I, L). Control larvae have clearly defined interray zones (black asterisks) and distal ray regions (black arrow) (D-E, G-H, J-K). Inta11: NTR + MTZ larvae have poorly defined interray zones (red asterisks) (F, I, L) similar to less developed rays in control larvae (red asterisks) (D-E, G-H) and the distal tips of the fin rays are nearly indiscriminate from the surrounding regions of the fin fold (F, I, L). Inta11: NTR + MTZ larvae show premature proximal fin ray calcification (purple asterisks) in the anterior rays (F, I, L) which is absent in control larvae (D-E, G-H, J-K). Estimate of fin ray length reduction highlighted with dotted line (J-L). As distal tip of fin rays are undefined in Inta11: NTR + MTZ, length measurements were not possible. Dotted lines span from proximal edge of fin ray to the distal edge of the fin fold at fin rays 2, and 3 (L). Identical length lines are then superimposed over control fin rays 2 and 3 (J, K) to show discrepancy in length. Disc matrix decomposition has started between presumptive proximal radials 1 and 2 (yellow asterisks) (D-F) at 6.8mm in length, and presumptive proximal radials 3 and 4 (yellow asterisks) at 7.2mm in length (G-I). All Inta11: NTR + MTZ larvae are 30dpf, and staged matched control larvae were selected based on three different staging criteria (standard length, spinal cord/rib calcification progress, and stages of endoskeletal disc cartilage matrix decomposition). Scale bars: 100μm in A-L.

In order to observe the effects of fin fold mesenchyme ablation on the development of lepidotrichia (fin ray), we established a protocol for rearing zebrafish larvae in metronidazole until 30dpf (S1 Fig). We initially proposed examining the formation of caudal fin rays as these were observed as early as 12dpf in treated *Tg(Inta11:NTR)* individuals. Following a “juvenile 4” treatment (S1 Fig), *Tg(Inta11:NTR)* larvae did not show any caudal fin ray defects at 20dpf (n = 4, 7.4% survival rate (S1 Fig)). As the defects observed at 7dpf for pectoral fin appeared more pronounced than those in the median fin at the same stage (Fig 4), we decided to extend the treatment to observe the effects on pectoral fin ray formation. Due to developmental delays in treated *Tg(Inta11:NTR)* larvae (S3 Fig), we decided to raise this group of fish until 30dpf, when fin ray formation was initiated. “Juvenile 5” treatment was devised in order to decrease rates of mortality in the MTZ-treated group (S1 Fig). Following alcian blue and alizarin red staining for cartilage and bone respectively, zebrafish were staged based on three criteria: standard length, cartilaginous disc decomposition, and calcification of spinal cord and ribs (Fig 8 and S3 Fig). At 30dpf, all treated *Tg(Inta11:NTR)* larvae have pectoral fin defects (n = 7) (Fig 8C, 8F and 8I), and the three most developed larvae display pectoral fin ray defects (n = 3) (Fig 8F and 8I). At developmental stage 6.4mm, MTZ-treated *Tg(Inta11:NTR)* larvae continue to display fin fold collapse and actinotrichia defects (n = 4) (Fig 8C) compared to stage-matched controls (Fig 8A and 8B). Initial formation of the anterior most rays does not appear delayed in 6.4mm treated larvae compared to controls (Fig 8A–8C). At length 6.8, and 7.2mm, treated larvae show several key differences with control larvae: the larvae have two missing posterior rays at each stage (5, and 6 compared to 7, and 8 respectively). The rays are shorter (Fig 8L) and less distally defined; the interray zones are poorly defined, and the proximal fin ray regions show premature calcification in the anterior most rays (Fig 8F, 8I and 8L). Control larvae have longer fin rays (Fig 8J and 8K) that have distinct distal tips, with clearly defined interray zones (Fig 8D, 8E, 8G, 8H, 8J and 8K). No calcification is observed in any control larvae at these developmental stages (Fig 8D, 8E, 8G, 8H, 8J and 8K). The poorly defined interray zones of treated larvae (Fig 8F, 8I and 8L) are comparable to the less developed posterior fin rays in the control larvae (Fig 8D, 8E, 8G, 8H, 8J and 8K). By comparing treated *Tg(Inta11:NTR)* larvae at 7.2mm (Fig 8I and 8L) to less developed control larvae (WT + MTZ, 6.8mm; untreated, 6.9mm) (Fig 8D and 8E), we can remove the possibility that these defects are simply due to a delay in fin ray development. At 30dpf, treated transgenic larvae continue to show no defects in caudal fin rays (n = 7, 13.5% survival rate (S1 Fig) compared to controls (S3 Fig). However, a small percentage of larvae in each treatment group display unrelated major caudal fin deformities (S3 Fig). The number of fish with caudal fin deformities is displayed in the bottom right corner of each panel and is presented as a fraction over total observed fish (S3 Fig). These results show that a sustained daily metronidazole treatment can sufficiently ablate the fin fold mesenchyme to produce pectoral fin ray defects at 30dpf. Thus, we are able to show that defects in larval fin fold mesenchyme, through genetic ablation, can produce truncated dermal bone elements in adult pectoral fins.

## Discussion

### Implications for the fin-to-limb transition

We have shown that, following metronidazole treatment, we can consistently and efficiently ablate fin fold mesenchyme in *Tg(Inta11:NTR)* larval zebrafish. Furthermore, the apoptotic nature of cell ablation was confirmed via TUNEL assay. This population of cells has been shown to express multiple 5’*hox* genes, including *hoxa13a*, *hoxa13b*, *hoxd13a*, and *hoxa11b* [30] and *actinodin* genes [22–23]. Enhancer, cell lineage-tracing and knockout data have also proposed a deep homology between fin fold mesenchyme in teleost and presumptive autopod mesenchyme of tetrapods [33–35, 38]. In fact, fin mesenchyme migration failure has been proposed as a mechanism of fin dermal bone loss during limb evolution [30, 33]. Actinotrichia are crucial for proper fin fold mesenchyme migration, and thus seem to be an obvious target to disrupt migration [19, 22, 24]. We have previously explored the possibilities of *actinodin* loss/change in regulation as mechanisms of actinotrichia defects and subsequent loss of dermal bone in fins of tetrapods [22–23].

Our data show that fin fold mesenchyme survival is crucial for fin fold and actinotrichia maintenance. Following ablation, the larval median and pectoral fin folds collapse and disorganized actinotrichia are observed. The actinotrichia are no longer able to support the fin fold. They are unable to remain rigid, and bend parallel to the collapsed fin fold region (Fig 5). It appears that fin fold mesenchymal cells contribute to their own successful migration, likely through their contributions to structural components of the actinotrichia [19, 22–23]. Indeed, we show that *and1* reporter expression in the mesenchyme is decreased using the *Tg(2PΔEpi:mCherry)* transgenic line, demonstrating lower *and1* activity (Fig 7G–7J). At the same time, previous studies have also proposed that *hoxa13* contributes to pectoral fin fold formation [18]. Thus, *hoxa13a/hoxa13b* double mutants display a reduced larval pectoral fin fold size [33]. Although *Tg(Inta11:NTR)*larvae do show reduced transcripts for *hoxa13a*, levels of *hoxa13b* do not appear to be affected, suggesting the mechanisms of fin fold reduction (ie. actinotrichia defects) differ from that occurring in *hoxa13a/hoxa13b* double mutants. No fin fold defects are observed in single mutant larvae (*hoxa13a -/*- or *hoxa13b -/*-) nor in *hoxd13a* mutant larvae [33]. In addition, despite similar reductions in size, pectoral fin fold morphology appears to differ in *hoxa13a/hoxa13b* double mutants compared to *Tg(Inta11:NTR)*larvae following ablation. The pectoral fin folds of treated *Tg(Inta11:NTR)*larvae show a characteristic distal “peak” as the actinotrichia bend (Figs 4 and 5), which is absent in *hoxa13a/hoxa13b* double mutants. Finally, no effect is described in the median fin fold of *hoxa13a/hoxa13b* double mutants [33]. Additional work is required to investigate the contributions of *5’hox* to the larval fin fold.

To assess the effects of prolonged fin fold mesenchyme ablation on later larval fin ray formation, we developed a protocol to treat the larvae until 30dpf. Through sustained metronidazole treatments, we were able to show pectoral fin ray defects at 30dpf (6.8–7.2mm) in *Tg(Inta11:NTR)*larvae. These larvae have lower fin ray numbers, which are shorter and less distally defined compared to stage-match control larvae (Fig 8). Fin fold mesenchyme has been shown to contribute to both fibroblast and osteoblast lineages [33, 37]. To assess the contributions of fin fold mesenchyme Nakamura et al. used a “late-phase” *hoxa13* enhancer to drive the Cre recombinase enzyme in their lineage-tracing experiments. Since the *lnta11* regulatory element used in the present study is activated by Hoxa13a & Hoxd13a, fin fold mesenchymal cells that show *Inta11:NTR* transgene expression would also contribute to the osteoblast lineage. We propose a loss or disorganization of osteoblasts can account for the mispatterning of the fin rays in *Tg(Inta11:NTR)* larvae following metronidazole treatment. Proteoglycans are known to be involved in the organization of endochondral and intramembranous bone extracellular matrix. They actively regulate collagen fibrillogenesis and are secreted by differentiating and mature osteoblasts [48–49]. In *Tg(Inta11:NTR)* fish, alcian-blue stained proteoglycans appear dispersed through the adult fin fold rendering the interray tissue and distal fin ray regions undefined (Fig 8F, 8I and 8L). In control larvae, the presumptive fin ray definition appears to be due to condensation of alcian blue-stained proteoglycans (Fig 8D, 8E, 8G, 8H, 8J and 8K). An analysis of osteoblast markers is necessary to provide conclusive evidence for a loss or disorganization of osteoblasts, however due to limited sample number, further analysis was not possible. These results provide evidence that larval fin fold mesenchyme defects can produce a truncation in late larval pectoral fin rays and may have potential implications for the loss of fin dermal bone in tetrapods.

In contrast to pectoral fin ray defects, no caudal fin ray defects were observed (S3 Fig). We showed that at 7dpf, median fin fold defects are ameliorated compared to 72hpf in *Tg(Inta11:NTR)* following metronidazole treatment suggesting an early reversal of the severe larval median fin fold phenotype. We propose several explanations for this observation. First, transgenic zebrafish frequently display variable transgene expression between individuals and this may have resulted in inconsistent ablation [50]. This contingency, combined with higher relative numbers of fin fold mesenchymal cells in the median fin (Fig 1F and 1J), could result in a reduced effect in the median fins compared to the pectoral fins. Second, in order to avoid metronidazole toxicity at 72hpf, “larval1” treatment spanned from 30-60hpf and may have missed a key developmental period from 60-72hpf important for long-term median fin fold maintenance. Finally, fin fold mesenchyme ablation may simply yield a more severe and prolonged effect in the pectoral fin due to differences in patterning and morphology. In fact, transitional tetrapods maintained caudal fin rays for millions of years following the loss of dermal bone in paired appendages [51] suggesting the caudal fin may have been less susceptible to the mechanisms of fin dermal bone loss ie. fin fold mesenchyme defects.

### Implications for fin development

Following fin fold migration defects, a shift in cell fate from dermal to endochondral bone progenitors has been proposed as a mechanism for simultaneous dermal reduction and endochondral expansion [33]. We have shown dermal bone reductions following fin fold mesenchyme ablation at 30dpf; however no effects are observed in the presumptive proximal and distal radials (Fig 8). We recognize that cell ablation methods are not ideal to test any hypotheses of fin fold mesenchyme shifts in cell fate. We are not differentially allocating these cells to the proximal regions of the pectoral fin, we are simply lowering the number of cells that properly migrate and contribute to adult dermal bone. We therefore do not predict an expansion of endochondral bone, simply a reduction in fin dermal bone (fin ray defects).

*Tg(Inta11:NTR)*larvae show decreased endoskeletal disc size at 7dpf following metronidazole treatment, which is in contradiction with a hypothesis of endochondral bone expansion during limb evolution [14, 33, 52]. We propose that disc size reductions are a secondary effect of the fin fold mesenchyme ablation. As shown by *in situ* hybridization, lower amounts of distal transcripts are present for *hoxa13a*, and *hoxd13a* (Fig 7A–7F). While *hoxa13b* and *hoxa11b* are expressed in the ablated population of cells, their expression patterns extend much more proximally in the pectoral fin, outside the zone of ablation. This may account for the absence of obvious decreases in distal expression, as many *hoxa13b/hoxa11b*-expressing cells are not affected (S2 Fig). Overall, we provide evidence for a global decrease in 5’*hox* transcripts, which has been linked to decreased FGF signalling and endochondral bone size. Decreased FGF signalling between the apical ectodermal ridge and underlying limb mesenchyme results in reduced endochondral bone during limb development in mice [11, 46, 47]. We show decreased levels of transcripts for *fgf10a* (Fig 7O–7R), however levels of *fgf8a* transcripts are unaffected (S2 Fig). Lower levels of *fgf10a* expression may be due to ablation of *fgf10a*–expressing cells or lowered 5’Hox signalling. Finally, the change in expression of *shha* and *ptch2* highlight the reduced endoskeletal disc proportions (Fig 7K–7N). Altered *shha* expression may be due to lower transcript levels of *fgf10a* or simply an effect of the disc size reduction itself. Feedback loops between 5’Hox, FGF and HH signalling are well-documented [42, 44–46].

In contrast to decreased presumptive endochondral bone elements at 7dpf, *Tg(Inta11:NTR)*larvae show earlier dermal bone calcification in the proximal regions of the anterior-most fin rays at 30dpf following metronidazole treatment. This suggests increased osteoblast activity in this zone. At 30dpf, we show high levels of YFP-expressing cells in the proximal fin fold mesenchyme and anterior-most fin rays in the pectoral fin (S4 Fig), indicative of sustained or reactivated *hoxa13a/hoxd13a* expression in these regions. We have previously shown that *m-Inta11* regulatory element is activated by Hoxa13 and Hoxd13 in mice [32]. We propose that increased osteoblast activity may be due to altered FGF-signalling in response to the reactivation of *hoxa13a* /*hoxd13a* during fin fold mesenchyme regeneration. An analysis of osteoblast markers is necessary to provide conclusive evidence for increased osteoblast activity, however due to limited sample number, further analysis was not possible. *5’Hox* signalling has been shown to contribute to *Fgf10* signalling in mice [42]. In addition, FGF-signalling has been linked to the induction of osteoblast/chondrocyte differentiation, and the promotion of intramembranous bone ossification [53–55].

### Nitroreductase/Metronidazole system for long-term cell ablation

The nitroreductase/metronidazole system was devised as a tool for analysing developmental and regenerative processes following cell-specific ablation [39]. Unfortunately, the regenerative capabilities of zebrafish fins make long-term sustained cell ablation difficult. We, and others, show that the NTR/MTZ system is efficient at specifically ablating cells of interest in short-term experiments [40]. At 3dpf, we observed nearly complete fin fold mesenchyme ablation in the median fin fold. If metronidazole is removed however, a secondary wave of YFP-expressing fin fold mesenchyme appears within 48 hours (S4 Fig). To assess the effects of fin fold mesenchyme ablation on fin ray formation, we therefore had to maintain metronidazole exposure during the entire course of development. At high concentrations or following prolonged exposure times, metronidazole is toxic and can induce non-specific effects [40]. We therefore utilised a more efficient triple mutant variant of nitroreductase in order to reduce treatment length/concentration and minimize metronidazole toxicity [40]. Furthermore, to promote survival of larvae, metronidazole concentrations were reduced as development progresses and 6 hour rotifer baths/feeding breaks were provided daily (S1 Fig). Despite all these measures, *Tg(Inta11:NTR)* larvae had extremely low survival rates (13.5%) at 30dpf (S1 Fig) and experienced severe developmental delays (S3 Fig) following a “juvenile 5” treatment. No caudal fin ray defects were observed in the surviving larvae and only modest pectoral fin ray defects were produced. High levels of YFP-expressing cells were present in the caudal fin and in the proximal regions of the pectoral fins of MTZ-treated *Tg(Inta11:NTR)* larvae at 30dpf. These observations highlight regeneration of the fin fold mesenchyme despite the sustained metronidazole treatment (S4 Fig). “Juvenile 4” and juvenile 3” treatments, with minimal increases in metronidazole concentration, result in more severe developmental delays and complete lethality of *Tg(Inta11:NTR)* larvae prior to 30dpf (S1 Fig). WT larvae exposed to metronidazole also show impaired survival following a “juvenile 3” treatment (S1 Fig). In summary, it seems the metronidazole concentrations required for survival *Tg(Inta11:NTR)* larvae to 30dpf are insufficient to maintain complete fin fold mesenchyme ablation. Therefore, metronidazole toxicity and the regenerative properties of zebrafish decrease the usefulness of this system for long-term cell ablation of *hoxa13a/hoxd13a*-expressing fin fold mesenchyme. The difficulties of raising zebrafish in metronidazole are worsened by the existence of an additional secondary pattern of YFP-expressing cells in the lower digestive tract of *Tg(Inta11:NTR)*larvae (S1 Fig). These cells do express *hoxa13a*, as confirmed by whole mount *in situ* hybridization (S1 Fig), and are therefore also subject to cell ablation. The ablation of *hoxa13a*-expressing cells in the lower digestive tract likely contributed to developmental delays and decreased survival of MTZ-treated *Tg(Inta11:NTR)* larvae.

In conclusion, we show that ablation of fin fold mesenchyme results in actinotrichia defects and collapse of the median and pectoral fin folds in zebrafish larvae. Using a sustained 30 day metronidazole treatment, we are also able to produce pectoral fin ray defects. We propose that the ablation of mesenchymal cells results in a failure to maintain the actinotrichia, likely due to a decrease in total actinodin proteins, and subsequent collapse of the fin fold. Impaired migration of any surviving or regenerating fin fold mesenchyme results in a lower number of presumptive osteoblasts that are also less organized, thus creating defects in fin ray length, number and definition. In addition, 7dpf larvae show a reduction in endoskeletal disc size, while 30dpf larvae show earlier calcification in the proximal regions of the fin rays. We propose that these phenotypes are long term effects from the initial cell ablation and subsequent regeneration.

## Methods

### Animal care

All fish are bred and raised in the D’ Iorio, University of Ottawa, zebrafish facility. Wild-type zebrafish stock has been bred in the laboratory for several years. The fish facility is maintained at 28°C, with a photo-period of 14 h of light and 10 h of darkness [56]. Animal care and experiments were certified by the Canadian Council on Animal Care and licensed under the Ontario Animals for Research Act. Zebrafish larvae were anesthetized and euthanized using tricaine.

Approved protocol number: BL-1851.

### Plasmid construction

All cloning and subcloning was performed following standard procedures [57]. The original vector used was *pEGFP-N1*. The CMV regulatory regions were removed and Tol2 arms were inserted between the *AseI* and *NheI* (left arm) and *AflII*(right arm).

The human beta-globin minimal promoter was isolated from the *p1230* vector, amplified, and inserted in the BamHI, and AgeI sites using the following primers:

**FW** 5’ GGATCCCTGGGCATAAAAGTCAG 3’

**Rev** 5’ ACCGGTTCTGCTTCTGGAAGGCT 3’

m-Inta11 element was inserted at the XmaI site according to [32].

The YFP-NTR transgene was amplified from the pBK 2xNRSE-2xzHB9-5xUAS-TagYFP-T2A-NTR plasmid and inserted into the AgeI and NotI sites using the following primers:

FW 5’ ACCGGTATGGTTAGCAAAGGCGAGG

Rev 5’ GCGGCCGCTTACACCTCTGTCAGGGTGA

### Microinjection in Zebrafish embryos and transgenic Zebrafish

Constructs (final concentration of 100ng/μL) are co-injected with transposase RNA (final concentration of 50ng/μL) mixed with RNAse-free water and 0.5% phenol red in one cell-stage zebrafish embryos.

Transgenic lines were identified via fluorescence microscopy and the expression patterns were confirmed with 3 lines. The transgenic line with the brightest YFP expression was used for experiments.

### Treatments

For “Larval 1–3” treatments (S1 Fig), larvae were raised in petri dishes with 50 embryos in 25ml of treatment solution (E3 media + MTZ/1%DMSO). For “larval 3” treatment (S1 Fig), solutions were changed daily and larvae were raised in the incubator with no light cycle. Larvae were not fed. Larvae displaying non-specific defects at 7dpf from MTZ toxicity (heart edema, shortened trunk) were consistently omitted from analysis across treatment groups.

For “juvenile 1–5” treatments (S1 Fig), larvae were transferred to 1L tanks at 5dpf. Each 1L tank housed two ~450ml mesh bottom baskets containing 12–13 larvae. Mesh bottom baskets could be lifted and easily transferred to new 1L during treatment changes without harming the larvae. Treatment solution (Water + MTZ/1%DMSO) was changed daily, and larvae were raised at 28°C, with a photo-period of 14 h of light and 10 h of darkness [56]. Larvae were fed Gemma75 food once daily from 6-10dpf, twice daily from 10-15dpf, and Gemma150 four times daily from 16-30dpf.

For “juvenile 1–2” treatments (S1 Fig), one third of the treatment solution was a concentrated rotifer bath.

For “juvenile 3–5” treatments (S1 Fig), larvae were transferred to a rotifer bath (1/3 concentrated rotifers, 2/3 water) for 6 hours a day to facilitate feeding then returned to the treatment solution (Water + MTZ/1%DMSO). From 16dpf onward, larvae were simply transferred to water for 6 hours daily instead of the rotifer bath.

All treatments are made with 1% DMSO.

### TUNEL assay

Embryos were fixed in 4%PFA O/N at 4°C. Following rehydration in PBST, embryos were permeabilized by digestion with 25mg/μl proteinase K in PBS for 20min at RT. Embryos were then post-fixed for 20min in 4%PFA and washed 5X in PBST, 5 min each wash. Following washes, embryos were permeabilized using fresh 0.1% sodium citrate in PBST for 15min at RT, and then washed 3X in PBST, 5 min each wash. TUNEL reaction mix was added according the manufacturer’s instructions (Roche Cat#12156792910) and incubated for 2hours at 37°C in the dark. Following TUNEL reaction, embryos were washed 3X in PBS for 5 min each wash.

### *In situ* hybridization

*In situ* hybridization on whole-mount embryos was performed as previously described [58]. Digoxigenin-labelled antisense RNA probes were generated using the following cDNAs: *and1* (2,383 base pairs (bp) [22], *hoxd13a*(793bp, [22]), *shha* (2.5kb, [59]), *fgf10a*(1.6kb; kindly provided by I. Belmonte), *fgf8a*(1.5kb; [22]), *hoxa13a*(500 bp; Addgene 36463, [30]), *hoxa13b* (700 bp; Addgene 36568, [30]), *hoxa11b* (800 bp; Addgene 36466, [30]), *ptch2*(1.15 kb; [60]) and *hhip*(2 kb;).

*hhip* cDNA was amplified using the following primers

FW 5’ATGAAGCATTTGAAATTTGTGCT

Rev 5’GTCTTTCTCACCGTCCCCTT

### Immunohistochemistry (IHC)

Embryos were fixed in 4%PFA O/N at 4°C, and then stored in methanol. From methanol, larvae are permeabilized in acetone at -20°C for 20 min. No proteinase K treatment is required. Following 2X5min washes in PBST, larvae are placed in blocking solution for 3 hours (10% calf serum, 0.5% TritonX100 in PBS) and then incubated in primary antibody overnight (Primary Antibody: mouse anti-Collagen II (II-II6B3, 1:100 dilution in blocking, Developmental Studies Hybridoma Bank). Following 4X10min PBST washes, larvae are incubated in secondary antibody (Secondary Antibody: Alexa Fluor Goat anti-Mouse 488, 1:500 in PBST, Life technologies) for 3 hours at room temperature. Larvae are then washed 4X10min in PBST, with the first wash containing DAPI (1:10 000) stain.

### Transgenic fish

*Tg(Kr19)*transgenic line

Transgene is integrated 32,151 bp downstream of ENSDARG00000078279, and at a second unknown integration site. Kr19 fish show membrane-tethered KillerRed expression in the choroid plexus and endoskeletal disc cells, among other regions [61].

### Bone and cartilage staining

Following O/N fixation in 4%PFA at 4°C, 20-30dpf larvae were stained using the “Two-color acid-free” method previously described [62]. 60mM MgCl_2_ was used for alcian blue stain.

### Fin measurements

Adobe Photoshop CS6 was used to take the fin measurements. Each fin is measured 5 times, and the 3 median values are then averaged for a single value per fin. 7dpf median fins and pectoral fins were dissected off and imaged using the stereoscopic microscope. Median fin photos from 48-72hpf were taken whole-mount using the dissection scope. The position of migrating cells in the median fin fold is visualized using highly contrasted brightfield images. The distal most cell is used to measure the distance migrated. The pectoral fin was precluded for cell migration measurements as these cells are not visible without fin dissection. Fin mounting further creates difficulties regarding mesenchymal cell visibility due to their subsequent flattened morphology.

## Supporting information

S1 Fig Schematic of larval and juvenile treatments, including survival rate of “juvenile 3–5” treatments. **(A-B)** All metronidazole treatments tested, including survival rates in “juvenile 3–5” treatments. **(C)** Secondary expression pattern of *hoxa13a*, YFP-NTR in digestive tract. “Larval 1–3” treatments used for all stats in Fig 6 (A). “Juvenile 1–2” treatments resulted in completely lethality at 13dpf of all treatment groups (A). “Juvenile 3–5” treatments included 6-hour daily breaks (A), however only “juvenile 5” treatment resulted in some Inta11: NTR + MTZ survival (13.5%) by 30dpf (B). WT + MTZ and Inta11: NTR—MTZ showed no difference in survival rate at 20dpf using “juvenile 5” treatment (B), and larvae were not raised to 30 dpf as they developed faster than Inta11: NTR + MTZ. Secondary expression pattern of NTR in digestive tract at 7dpf (white arrow) (D), consistent with *hoxa13a* expression during early larval development (black arrow) (C). Digestive tract YFP-NTR expression maintained throughout late larval development (25dpf) (white arrow) (E-F). Brightfield (C, E), fluorescence (D, F).
(TIF)

S2 Fig *A*ltered gene expression profiles in the median and pectoral fin of *Tg(Inta11:NTR)* larvae following metronidazole treatment. **(A-J’)** Whole-mount *in situ* hybridization data showing altered, and unaltered gene expression profiles in the median and the pectoral fins of WT + MTZ, Inta11: NTR—MTZ, and Inta11: NTR + MTZ larvae. Inta11: NTR + MTZ larvae show altered gene expression patterns for *hoxa13a*, *hoxd13a*, *shha*, *ptch2*, and *fgf10a* as indicated in Fig 6 (A-R, H’-J’). WT—MTZ+DMSO larvae are included (A, D, G, J, M, P, H’). Inta11: NTR + MTZ show no difference in gene expression for *hoxa13b*, *hoxa11b*, *and1*, *hhip*, and *fgf8a* in the pectoral fin at 72hpf compared to control larvae (S-G’). Probe is indicated in the top right corner of each panel in the 1^st^, and 4^th^ column (A, D, G, J, M, P, S, V, Y, B’, E’, H’), age is indicated in the top right corner of each panel in the 3^rd^, and 6^th^ column (C, F, I, L, O, R, U, X, A’, D’, G’, J’). Number of larvae displaying gene expression pattern is indicated in the bottom right corner of each panel (A-R, H’-J’). Probes with no difference in gene expression do not have a value for number of larvae (C-G’), however each *in situ* hybridization experiment had 10–15 larvae per treatment group. Scale bars: 100μm in A-C, H’-J’; 50μm in M-R, V-X, B’-G’; 30μm in D-L, S-U, Y-A’.
(TIF)

S3 Fig Absence of caudal fin defects in *Tg(Inta11:NTR)*larvae at 30dpf following metronidazole treatment, small percentage of larvae display major unrelated caudal fin defects in all treatment groups. Developmental delays in *Tg(Inta11:NTR)*larvae following metronidazole treatment. **(A-C)** Whole-mount view of larvae used for Fig 8G–8L. **(D-F)** Example of major caudal defects present in all treatment groups. **(G-I)** Comparison of larvae development between Inta11: NTR—MTZ and Inta11: NTR + MTZ. At 30 dpf, Inta11: NTR + MTZ larvae do not show caudal fin ray defects (black arrow) (A) compared to control larvae (black arrows) (B-C). Calcification of entire spinal cord and first 3–4 ribs (red arrow) used for stage matching between treatment groups. Standard length present in bottom right corner (A-C). Pectoral fins were dissected and imaged for Fig 8G–8L. All treatment groups (WT + MTZ, Inta11: NTR—MTZ, Inta11: NTR + MTZ) have a small percentage of larvae with major unrelated caudal fin defects (Yellow arrow) (D-F). Number of larvae with phenotype present in bottom right corner of each panel (D-F). Inta11: NTR + MTZ larvae show >5 day developmental delay at 20dpf (I). Inta11: NTR—MTZ larvae at 15dpf (G), and 20dpf (H) shown as comparison. Beginning of caudal fin ray formation detected in 15dpf Inta11: NTR—MTZ (G), and 20dpf Inta11: NTR + MTZ larvae (I) (Green arrow). All caudal fin rays present at 20dpf in Inta11: NTR—MTZ larvae (blue arrow) (H). Scale bars: 50μm in A-C, D-F, H; 30μm in G, I.
(TIF)

S4 Fig Fin fold mesenchyme regeneration in *Tg(Inta11:NTR)*larvae following metronidazole treatment, incomplete ablation YFP-expressing cells in caudal and pectoral fin of *Tg(Inta11:NTR)* larvae at 30dpf. **(A-M)** Comparison of YFP-NTR expression in the median fin from 3-7dpf in Inta11: NTR—MTZ, I Inta11: NTR + MTZ, where treatment is halted at 3dpf, and Inta11: NTR + MTZ where treatment is maintained until 7dpf. **(N-O)** Levels of YFP-NTR expression in Inta11: NTR + MTZ at 30dpf. Inta11: NTR—MTZ larvae show highest levels of YFP-NTR expression in the median fin fold at 3dpf (white arrow) (A, D). YFP-NTR expression levels decline by 5dpf (white arrow) (B, E) and 7dpf (white arrow) (C, F) in Inta11: NTR—MTZ larvae. Following ablation, YFP-NTR expression is nearly absent in Inta11: NTR + MTZ larvae at 3dpf (red arrow) (G, J). If left untreated, a new wave of YFP-NTR-expressing cells are initiated in the proximal fin regions surrounding the trunk by 5dpf (green arrow) (H, K), and continue distal migration at 7dpf (green arrow) (I, L). If treatment is maintained in Inta11: NTR + MTZ until 7dpf, we continue to ablate YFP-NTR expressing cells (red arrow) (M). Note panel M is representative of “larval 3” treatment (S1 Fig), “juvenile 5” treatment requires 6-hour daily breaks and modified concentrations for larvae survival (S4 Fig). By 30dpf, Inta11: NTR + MTZ show high levels of YFP-NTR expression in both the caudal (N) and pectoral fin (O) indicating fin fold mesenchymal is constantly being regenerated. In the caudal fin, YFP-NTR expressing cells are along the entire proximal-distal length of the lepidotrichia (green arrow) (LP) and concentrated at the distal tip where the actinotrichia (AC) are present (green arrow) (N). In the pectoral fin, YFP-NTR expressing cells are only present in the proximal portions of the lepidotrichia and actinotrichia (green arrows), immediately adjacent to the endoskeletal elements (O). Border of the pectoral fin is highlighted by dotted line (O). Brightfield (A-C, G-I), fluorescence (D-F, J-O). AC, actinotrichia; EDE, Endoskeletal Elements; LP, lepidotrichia. Scale bars: 100μm in A-M.
(TIF)
