# Supplementary figures and images for: Effects of fin fold mesenchyme ablation on fin development in zebrafish
Source: PLoS One. 2018 Feb 8;13(2):e0192500. doi: 10.1371/journal.pone.0192500 (PMC5805328; doi:10.1371/journal.pone.0192500)

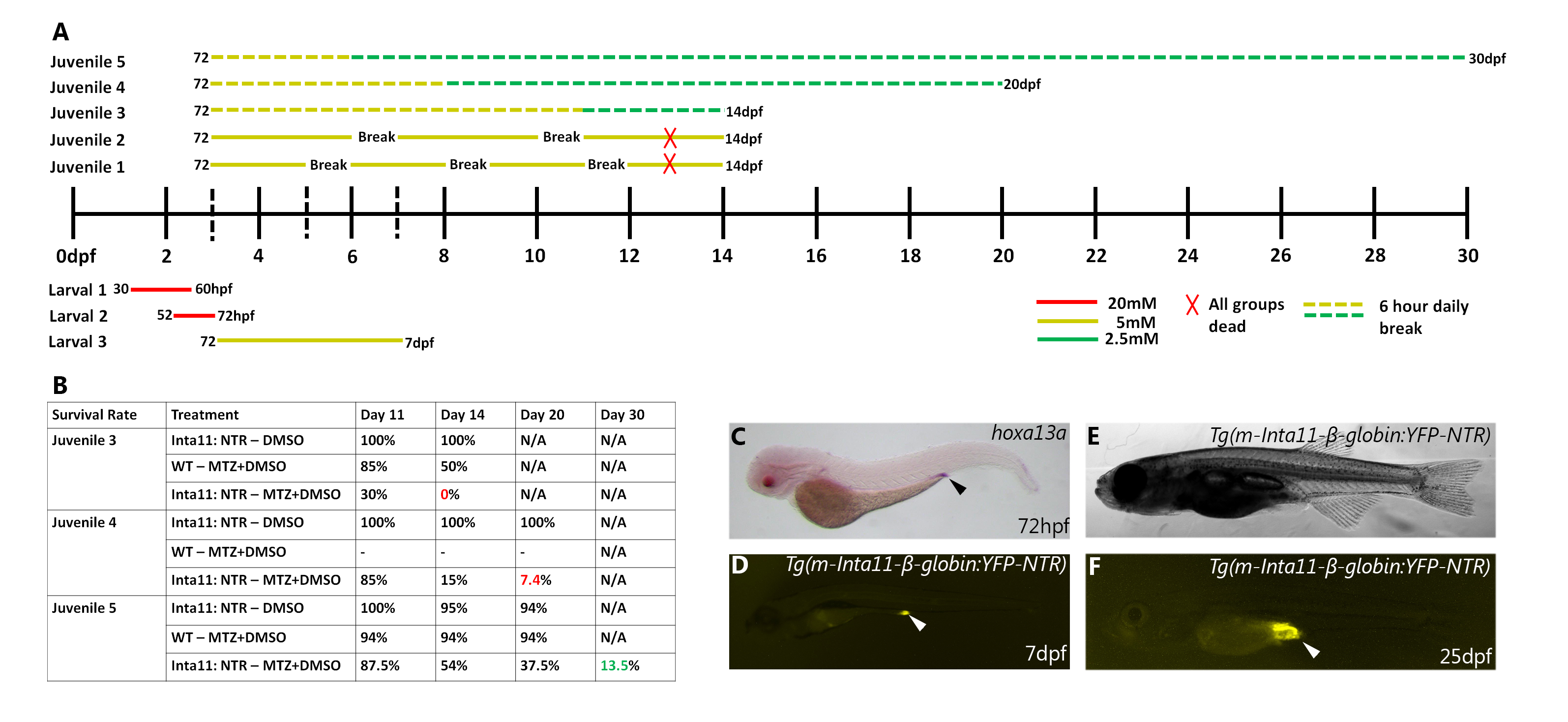

Supplement: S1 Fig — (A-B) All metronidazole treatments tested, including survival rates in “juvenile 3–5” treatments. (C) Secondary expression pattern of hoxa13a, YFP-NTR in digestive tract. “Larval 1–3” treatments used for all stats in Fig 6 (A). “Juvenile 1–2” treatments resulted in completely lethality at 13dpf of all treatment groups (A). “Juvenile 3–5” treatments included 6-hour daily breaks (A), however only “juvenile 5” treatment resulted in some Inta11: NTR + MTZ survival (13.5%) by 30dpf (B). WT + MTZ and Inta11: NTR—MTZ showed no difference in survival rate at 20dpf using “juvenile 5” treatment (B), and larvae were not raised to 30 dpf as they developed faster than Inta11: NTR + MTZ. Secondary expression pattern of NTR in digestive tract at 7dpf (white arrow) (D), consistent with hoxa13a expression during early larval development (black arrow) (C). Digestive tract YFP-NTR expression maintained throughout late larval development (25dpf) (white arrow) (E-F). Brightfield (C, E), fluorescence (D, F). (TIF) [file pone.0192500.s001.tif]

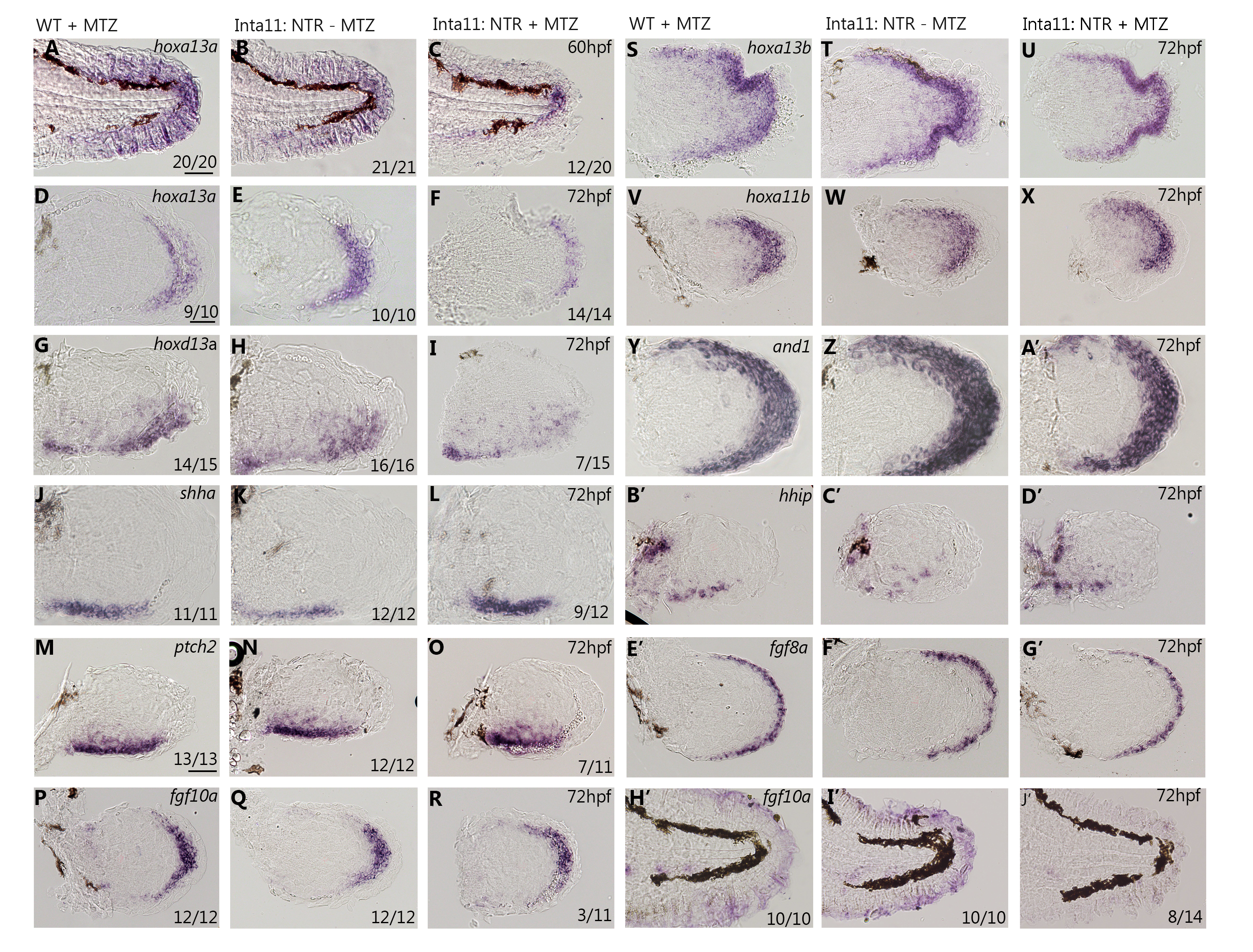

Supplement: S2 Fig — (A-J’) Whole-mount in situ hybridization data showing altered, and unaltered gene expression profiles in the median and the pectoral fins of WT + MTZ, Inta11: NTR—MTZ, and Inta11: NTR + MTZ larvae. Inta11: NTR + MTZ larvae show altered gene expression patterns for hoxa13a, hoxd13a, shha, ptch2, and fgf10a as indicated in Fig 6 (A-R, H’-J’). WT—MTZ+DMSO larvae are included (A, D, G, J, M, P, H’). Inta11: NTR + MTZ show no difference in gene expression for hoxa13b, hoxa11b, and1, hhip, and fgf8a in the pectoral fin at 72hpf compared to control larvae (S-G’). Probe is indicated in the top right corner of each panel in the 1st, and 4th column (A, D, G, J, M, P, S, V, Y, B’, E’, H’), age is indicated in the top right corner of each panel in the 3rd, and 6th column (C, F, I, L, O, R, U, X, A’, D’, G’, J’). Number of larvae displaying gene expression pattern is indicated in the bottom right corner of each panel (A-R, H’-J’). Probes with no difference in gene expression do not have a value for number of larvae (C-G’), however each in situ hybridization experiment had 10–15 larvae per treatment group. Scale bars: 100μm in A-C, H’-J’; 50μm in M-R, V-X, B’-G’; 30μm in D-L, S-U, Y-A’. (TIF) [file pone.0192500.s002.tif]

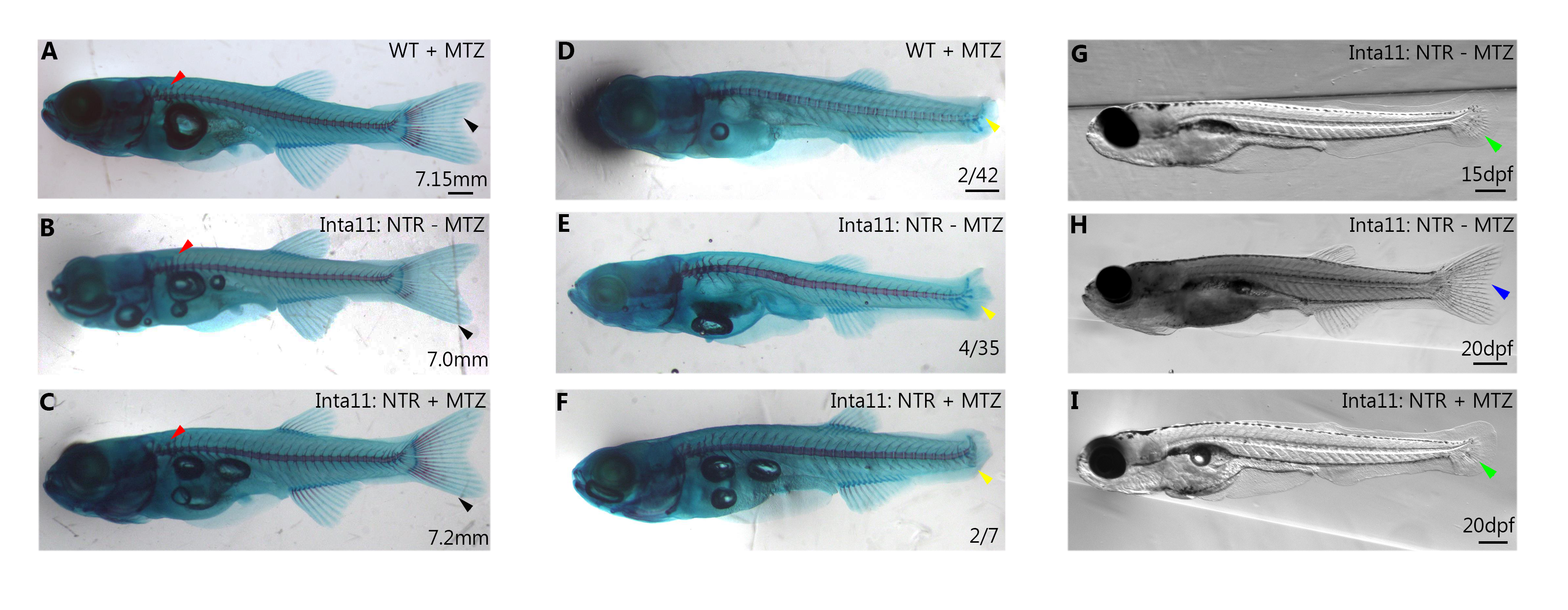

Supplement: S3 Fig — (A-C) Whole-mount view of larvae used for Fig 8G–8L. (D-F) Example of major caudal defects present in all treatment groups. (G-I) Comparison of larvae development between Inta11: NTR—MTZ and Inta11: NTR + MTZ. At 30 dpf, Inta11: NTR + MTZ larvae do not show caudal fin ray defects (black arrow) (A) compared to control larvae (black arrows) (B-C). Calcification of entire spinal cord and first 3–4 ribs (red arrow) used for stage matching between treatment groups. Standard length present in bottom right corner (A-C). Pectoral fins were dissected and imaged for Fig 8G–8L. All treatment groups (WT + MTZ, Inta11: NTR—MTZ, Inta11: NTR + MTZ) have a small percentage of larvae with major unrelated caudal fin defects (Yellow arrow) (D-F). Number of larvae with phenotype present in bottom right corner of each panel (D-F). Inta11: NTR + MTZ larvae show >5 day developmental delay at 20dpf (I). Inta11: NTR—MTZ larvae at 15dpf (G), and 20dpf (H) shown as comparison. Beginning of caudal fin ray formation detected in 15dpf Inta11: NTR—MTZ (G), and 20dpf Inta11: NTR + MTZ larvae (I) (Green arrow). All caudal fin rays present at 20dpf in Inta11: NTR—MTZ larvae (blue arrow) (H). Scale bars: 50μm in A-C, D-F, H; 30μm in G, I. (TIF) [file pone.0192500.s003.tif]

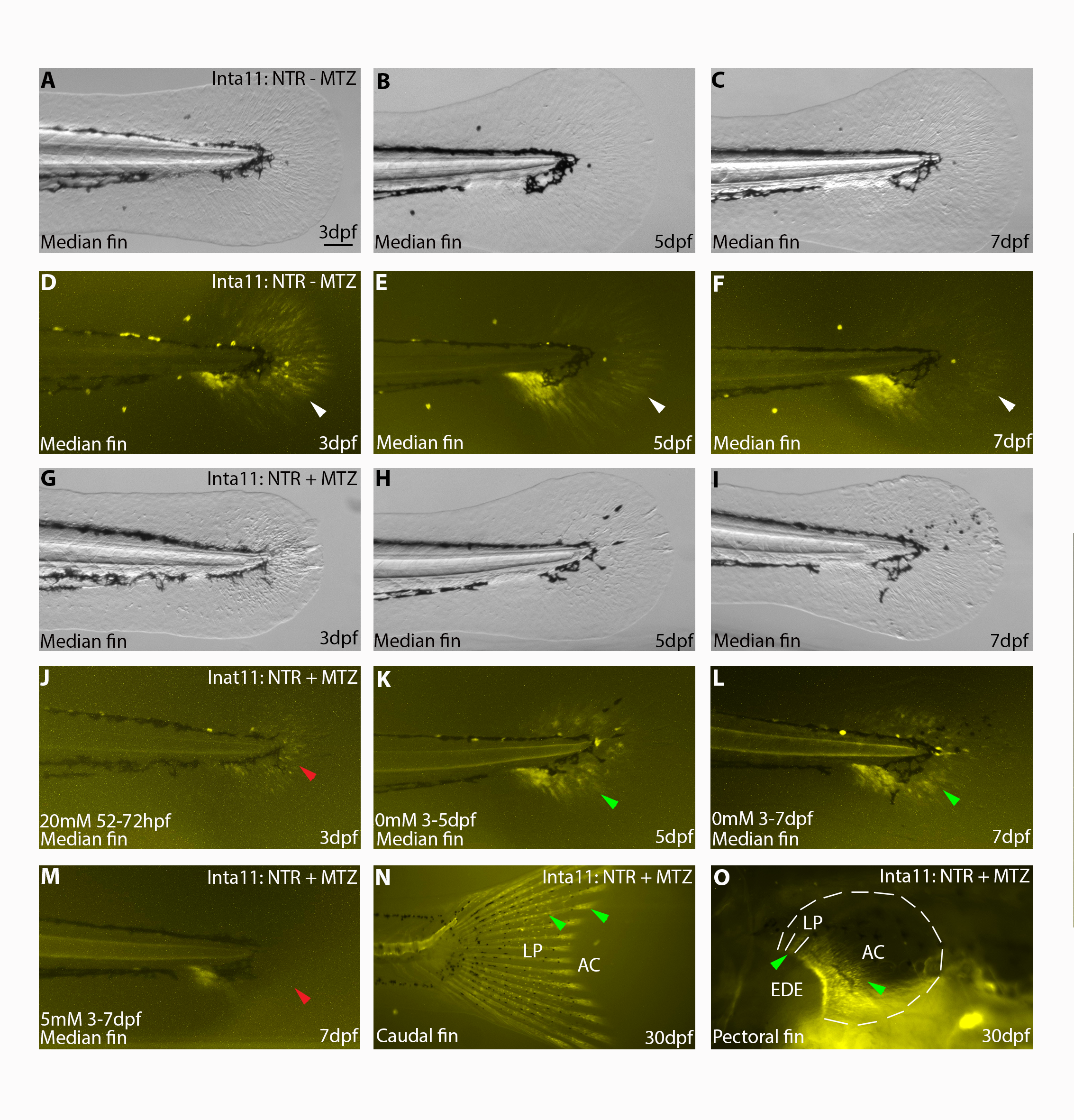

Supplement: S4 Fig — (A-M) Comparison of YFP-NTR expression in the median fin from 3-7dpf in Inta11: NTR—MTZ, I Inta11: NTR + MTZ, where treatment is halted at 3dpf, and Inta11: NTR + MTZ where treatment is maintained until 7dpf. (N-O) Levels of YFP-NTR expression in Inta11: NTR + MTZ at 30dpf. Inta11: NTR—MTZ larvae show highest levels of YFP-NTR expression in the median fin fold at 3dpf (white arrow) (A, D). YFP-NTR expression levels decline by 5dpf (white arrow) (B, E) and 7dpf (white arrow) (C, F) in Inta11: NTR—MTZ larvae. Following ablation, YFP-NTR expression is nearly absent in Inta11: NTR + MTZ larvae at 3dpf (red arrow) (G, J). If left untreated, a new wave of YFP-NTR-expressing cells are initiated in the proximal fin regions surrounding the trunk by 5dpf (green arrow) (H, K), and continue distal migration at 7dpf (green arrow) (I, L). If treatment is maintained in Inta11: NTR + MTZ until 7dpf, we continue to ablate YFP-NTR expressing cells (red arrow) (M). Note panel M is representative of “larval 3” treatment (S1 Fig), “juvenile 5” treatment requires 6-hour daily breaks and modified concentrations for larvae survival (S4 Fig). By 30dpf, Inta11: NTR + MTZ show high levels of YFP-NTR expression in both the caudal (N) and pectoral fin (O) indicating fin fold mesenchymal is constantly being regenerated. In the caudal fin, YFP-NTR expressing cells are along the entire proximal-distal length of the lepidotrichia (green arrow) (LP) and concentrated at the distal tip where the actinotrichia (AC) are present (green arrow) (N). In the pectoral fin, YFP-NTR expressing cells are only present in the proximal portions of the lepidotrichia and actinotrichia (green arrows), immediately adjacent to the endoskeletal elements (O). Border of the pectoral fin is highlighted by dotted line (O). Brightfield (A-C, G-I), fluorescence (D-F, J-O). AC, actinotrichia; EDE, Endoskeletal Elements; LP, lepidotrichia. Scale bars: 100μm in A-M. (TIF) [file pone.0192500.s004.tif]
